# Exploring the spatial interference effects elicited by social and non‐social targets: A conditional accuracy function approach

**DOI:** 10.1111/bjop.12735

**Published:** 2024-09-13

**Authors:** Renato Ponce, Juan Lupiáñez, Carlos González‐García, Maria Casagrande, Andrea Marotta

**Affiliations:** ^1^ Department of Psychology Sapienza University of Rome Rome Italy; ^2^ Department of Experimental Psychology & Mind, Brain and Behaviour Research Centre (CIMCYC) University of Granada Granada Spain; ^3^ Department of Dynamic and Clinical Psychology, and Health Studies Sapienza University of Rome Rome Italy

**Keywords:** conditional accuracy function, distributional analysis, gaze versus arrows, social attention, spatial interference effect

## Abstract

Recent studies employing the spatial interference paradigm reveal qualitative differences in congruency effects between gaze and arrow targets. Typically, arrows produce a standard congruency effect (SCE), with faster responses when target direction aligns with its location. Conversely, gaze targets often lead to a reversed congruency effect (RCE), where responses are slower in similar conditions. We explored this dissociation using the Conditional Accuracy Function (CAF) to assess accuracy across reaction time bins. Using a hierarchical linear mixed modelling approach to compare cropped eyes, and full faces as social stimuli, and arrows as non‐social stimuli, we synthesized findings from 11 studies, which led to three distinct models. The results showed that with non‐social targets, incongruent trials exhibited lower accuracy rates in the first bin than in subsequent bins, while congruent trials maintained stable accuracy throughout the distribution. Conversely, social targets revealed a dissociation within the fastest responses; alongside a general reduction in accuracy for both congruency conditions, congruent trials resulted in even lower accuracy rates than incongruent ones. These results suggest with gaze targets that additional information, perhaps social, in addition to the automatic capture by the irrelevant target location, is being processed during the earlier stages of processing.

The current debate in social attention research focuses on whether social orienting, which involves focusing on where others look or pay attention to, is influenced by general attentional mechanisms linked to stimulus directionality, such as eye‐gaze direction (Cole & Millett, 2019), or by rather specific social processes related to attributing intentions and mental states (Apperly & Butterfill, 2009). To explore this issue, researchers has recently employed the spatial interference paradigm (Cañadas & Lupiáñez, 2012; Jones, 2015; Marotta et al., 2018), using spatial Stroop‐like tasks to examine whether social stimuli can specifically trigger attentional processes.

The spatial Stroop task examines the conflict between processing task‐relevant information (i.e. direction of the stimuli), and irrelevant spatial information (i.e. physical location; see Lu & Proctor, 1995). In this task, participants must identify the direction of lateralized stimuli using lateralized responses while ignoring the spatial position. This set‐up results in congruent trials (e.g. arrows pointing left presented on the left side) and incongruent trials (e.g. arrows pointing left presented on the right side). According to Kornblum's (1992) taxonomy, the spatial Stroop produces a Type 8 ensemble, with three‐dimensional overlap between: (a) relevant and irrelevant stimulus dimensions, (b) relevant stimulus and the response dimensions and (c) irrelevant stimulus and response dimensions (see also Viviani et al., 2024).

Typically, non‐social stimuli produce faster responses in incongruent trials than incongruent ones, resulting in a standard congruency effect (SCE). According to multiple‐stage and multiple loci theories of Stroop interference (Banich, 2019; De Houwer, 2003; Parris et al., 2022), this phenomenon occurs because participants must address, during incongruent trials, the conflict at various levels, including task, stimulus and response.

Interestingly, Cañadas and Lupiáñez (2012) observed that participants were faster in incongruent trials when responding to the direction of lateralized gaze stimuli, producing a reversed congruency effect (RCE). This pattern was later replicated (Jones, 2015; Torres‐Marín et al., 2017) and compared with non‐social stimuli such as arrows (Marotta et al., 2018), and other social cues such as pointing hands (Bonventre & Marotta, 2023; Dalmaso et al., 2023), with the reversion occurring only with eye‐gaze. The RCE has also been observed with some animal faces (Ishikawa et al., 2024), inverted faces (Tanaka, Ishikawa, et al., 2023) and using oral responses (Narganes‐Pineda et al., 2022, experiment 3A). Intriguingly, Tanaka et al. (2024) observed the reversion using tongues as directional targets, proposing a dual‐route account, as described below.

To better understand the underlying mechanisms of this phenomenon, Marotta et al. (2019) conducted an electrophysiological study and found that early event‐related components (P1, N1 and N170) are similarly influenced by congruency in both eye‐gaze and arrow stimuli. However, opposite modulation patterns were observed in later components (N2 and P3). More recently, Narganes‐Pineda et al. (2023), in an fMRI study, observed common activations for eye‐gaze and arrow stimuli in right parieto‐temporo‐occipital regions during conflict resolution, alongside distinct activations between the frontal eye field (FEF) and occipital regions.

From a behavioural perspective, Román‐Caballero et al. (2021a, 2021b) found that variables reducing the SCE with arrows, such as the perceptual background complexity simultaneously increased the RCE for gaze trials. The necessary background segregation before selecting directional information created a temporal gap between processing relevant and irrelevant information. As a result, the irrelevant information decayed (*temporal‐delay hypothesis*; Hommel, 1993), reducing the spatial conflict, subsequently increasing the RCE observed for gaze targets. Hemmerich et al. (2022) found that arrows and words elicited a typical congruency sequence effect (see Gratton et al., 1992; i.e. decrease of the SCE after incongruent trials), whereas eye‐gaze and faces led to a similar increase of the RCE after incongruent trials. Importantly, the increase in the RCE was observed even when the arrow and gaze targets were presented mixed within the same block. Notably, RCEs increased with social, and SCEs decreased with non‐social stimuli following incongruent trials, regardless of the nature of the previous target. Additionally, a developmental study revealed that while a typical SCE was already observed in 4‐year‐olds and persisted throughout childhood for both eye‐gaze and arrows, the RCE only became evident for eye‐gaze stimuli from the age of 12 years (Aranda‐Martín et al., 2022).

Based on these observations, a recently introduced integrated framework for the RCE observed with gaze considers both domain‐general attentional processes and domain‐specific mechanisms (Hemmerich et al., 2022). Specifically, domain‐general attentional processes linked to the stimulus' pointing direction and its spatial location would produce similar spatial interference conflict for arrows and gaze, leading to faster responses for congruent conditions. In contrast, additional ‘special’ processes (e.g. the ‘looking vector’; Hemmerich et al., 2022) occur only with eye‐gaze, resulting in either slower responses for congruent or faster responses for incongruent gaze conditions, thus reversing the overall spatial conflict. Nevertheless, the exact ‘special’ mechanisms that modulate the interaction between task‐relevant and irrelevant information and are ultimately responsible for the RCE are still subject to ongoing debate.

Two main theoretical approaches have been explored to understand the phenomenon. The first emphasizes the importance of a social component. Initially, the eye‐contact hypothesis was proposed (Cañadas & Lupiáñez, 2012; Marotta et al., 2018), suggesting that participants would implicitly interpret the eyes in the incongruent condition (e.g. left eyes looking right) as looking at them, facilitating faster responses in incongruent trials. However, Narganes‐Pineda et al. (2022) demonstrated that explicit attention to the direction of eye‐gaze is essential for the RCE to occur, challenging the automaticity of the eye‐contact premise. Alternatively, Edwards et al. (2020) suggested that the RCE could be due to a joint attention episode (i.e. incongruent eyes would ‘look’ at fixation where participants are fixating). Despite this, the reversal was absent in children under 12 years (Aranda‐Martín et al., 2022), even though joint attention typically occurs before 4 years (Mundy et al., 2007). More recently, the joint distraction hypothesis was proposed (Hemmerich et al., 2022), claiming that congruent trials cause attention to shift away from the task, requiring a reorientation of attention and thereby slowing down reaction times. However, recent experiments have not supported this theory; Aranda‐Martín et al. (2023) used objects or external frames to prevent distraction, but the RCE was not modulated.

Despite the contrasting findings, there is still some evidence that supports the social interpretation of gaze underlying the reversed effect. The RCE seems influenced by the emotional expressions of faces (Jones, 2015; Marotta et al., 2022; Torres‐Marín et al., 2017), modulated by social familiarity (Ishikawa et al., 2024) and negatively correlated with social anxiety scores (Ishikawa et al., 2021).

Alternatively, a second theoretical approach suggests non‐social explanations based on either perceptual factors (Chen et al., 2022) or task‐related effects (Tanaka et al., 2024). For instance, Chen et al. (2022) observed that stimuli with equivalent perceptual features, regardless of their social versus non‐social nature, resulted in the SCE (although see Cañadas & Lupiáñez, 2012). This implies that congruency effects, whether elicited by eye‐gaze or arrow stimuli, are largely shaped by their perceptibility. Additionally, they suggested that variations noted in other studies could be attributed to changes in the breadth of attentional focus, as outlined by the zoom lens model of attention (Eriksen & St. James, 1986).

On the other hand, Tanaka, Oyama, et al. (2023) and Tanaka et al. (2024) introduced a dual‐stage theory, suggesting that a target background segregation and subsequent selective attention processes play a role in the reversion of the spatial congruency effect. According to this theory, the segregation of a target from its complex background delays the extraction of the target's directional information, as outlined by Román‐Caballero et al. (2021a, 2021b), allowing the location code to decay (Hommel, 1993). Following this segregation, selective attention acts to suppress the irrelevant spatial code, thereby resolving conflict (*activation‐suppression hypothesis*; Ridderinkhof, 2002a, 2002b). In the congruent condition, where the spatial code aligns with the directional code – such as when right‐looking gaze is presented in the right visual field – selective inhibition mechanisms suppress the correct spatial code (right), which delays the response. Conversely, in the incongruent condition, such as when a gaze directed to the right appears in the left visual field, selective inhibition suppresses the incorrect spatial code for the location (left), speeding up the correct response (right).

These two general frameworks' accounts (social vs. non‐social) are usually based on reaction time analyses, sometimes resulting in neglecting the analysis of accuracy rates (Dalmaso et al., 2023; Jones, 2015; Tanaka et al., 2024; Tanaka, Ishikawa, et al., 2023). However, although not always statistically significant, a dissociation in congruency effects between arrows and gaze has also been observed when considering error rates (Bonventre & Marotta, 2023, experiment 1; Cañadas & Lupiáñez, 2012, experiment 1; Ishikawa et al., 2021, experiment 1; Marotta et al., 2018, 2019).

Additionally, a consistent finding has been that participants generally make more errors in discriminating gaze direction than in discriminating arrows direction (Aranda‐Martín et al., 2023; Hemmerich et al., 2022; Ishikawa et al., 2024, experiments 3 & 4; Marotta et al., 2019; Narganes‐Pineda et al., 2022, experiment 3). Interestingly, 5‐year‐old children had higher accuracy rate with eye‐gaze than with arrows stimuli, while 12‐year‐olds had the opposite pattern (Aranda‐Martín et al., 2022).

Variations in accuracy have also been observed in studies using only faces as targets. For example, Torres‐Marín et al. (2017, experiment 1) reported lower accuracy rates for happy, angry and sad expressions in congruent trials compared to incongruent trials. Furthermore, lower accuracy rates with angry faces compared to other expressions have been also noted (Marotta et al., 2022; Torres‐Marín et al., 2017). Notably, Torres‐Marín et al. (2017, experiment 1) found that gelotophobes had higher error rates than non‐gelotophobes, whereas Marotta et al. (2022) observed that participants with low Autism Spectrum Quotient (AQ) scores made more errors with happy faces than fearful faces.

Considering the findings regarding accuracy and the importance of analysing the temporal dynamics highlighted in previous studies (Hemmerich et al., 2022; Marotta et al., 2019; Román‐Caballero et al., 2021a, 2021b; Tanaka et al., 2024; Tanaka, Oyama, et al., 2023), examining how accuracy varies over reaction time depending on the type of stimuli can be crucial for gaining insight into the dissociation observed between social and non‐social stimuli. Therefore, using a distributional approach (Balota & Abrams, 1995; De Jong et al., 1994), employing tools such as the Conditional Accuracy Function (CAF), could reveal when the congruency effects start to diverge between social and non‐social stimuli.

In conflict tasks, a distributional approach enables the examination of how experimental manipulations influence the conflict effect across different stages of cognitive processing (Mittelstädt & Miller, 2020; Rousselet et al., 2017). This approach allows for the exploration of the interaction between relevant and irrelevant information, based on a common theoretical framework that points to two parallel processing routes (Banich, 2019; Cohen et al., 1990; De Jong et al., 1994; Ridderinkhof, 2002a, 2002b; Ulrich et al., 2015). These dual‐route models propose that task‐relevant information is processed through controlled mechanisms, whereas irrelevant information is processed through more automatic, and short living mechanism.

Regarding the CAF, it correlates task execution time with accuracy (Heitz, 2014; Luce, 1986; Wood & Jennings, 1976), offering insights into how response accuracy varies with reaction time (van Maanen et al., 2019). Typically, with non‐social stimuli, accuracy is high across the distribution under congruent conditions but lower for the fastest reaction times under incongruent conditions, increasing as the RTs become longer (e.g. Hübner & Töbel, 2019; Torres‐Quesada et al., 2022; Ulrich et al., 2015). Within the dual‐route models, the initial boost of irrelevant spatial information by the automatic mechanism (Lu & Proctor, 1995; Torres‐Quesada et al., 2022; Ulrich et al., 2015) increases the likelihood of incorrect response in incongruent trials. However, across middle and slower RTs, as the automatic mechanism decreases, the controlled route enhances performance.

To date, no CAF analysis has used gaze targets. However, since modulations typically occur within the fastest responses, analysing this part of the distribution could reveal specific patterns. While electrophysiological evidence suggests that the dissociation in congruency effects between eye‐gaze and arrow stimuli occurs at later processing stages (Marotta et al., 2019), earlier stages might also influence this dissociation, especially considering that faces can be distinguished from other stimuli during early processing stages (e.g. Bentin et al., 1996; Nemrodov et al., 2014). Therefore, this early distinction might modulate the initial boost of the irrelevant spatial information.

Moreover, the temporal‐delay and active‐suppression hypotheses, which have been used to explain the RCE, were originally developed for the Simon effect (Hommel, 1993; Ridderinkhof, 2002a, 2002b). However, these hypotheses may not apply universally, as different mechanisms might operate across tasks (Funes et al., 2010; Liu et al., 2004; Pratte et al., 2010; Torres‐Quesada et al., 2014). Unlike the Simon task, the spatial Stroop involves stimulus–stimulus overlap (direction and location; see table 1 in Kornblum, 1994), which may influence temporal conflict facilitation and inhibition. Integrating our analyses with a spatial Stroop framework will help determine whether the observed effects are due to social components or task‐related phenomena.

Thus, in this study, the CAF tool was employed to analyse datasets from studies using the social variant of the spatial Stroop task (Marotta et al., 2018), with the goal of examining how accuracy rates vary over reaction time intervals and uncovering temporal dynamics underlying the dissociation between social and non‐social attentional mechanisms. Through this approach, we expect to explore hidden aspects not discernible through mean RT or error rates analysis alone, thereby contributing to a deeper understanding of the interplay between social and non‐social information processing.

## METHOD

### Studies

The studies included in this analysis are summarized in Table 1. Selection criteria focused on methodological similarities, specifically: (1) use of the spatial Stroop Task, (2) comparison between cropped‐eyes or faces as social targets and arrows as non‐social targets, (3) use of upright faces and (4) the implementation of an explicit discrimination task. Consequently, we excluded hand‐pointing targets (Bonventre & Marotta, 2023; Dalmaso et al., 2023), animal face targets (Ishikawa et al., 2024) and inverted faces (Marotta & Lupiáñez, 2018, unpublished manuscript; Tanaka, Ishikawa, et al., 2023). From Narganes‐Pineda et al. (2022), only data from the explicit task with motor responses (experiment 1) were included. We also incorporated studies using only faces to assess potential differences between cropped eyes and complete faces. These studies included emotional expression conditions with five levels: anger, happiness, sadness, fear and neutral (Marotta et al., 2022; Torres‐Marín et al., 2017).

**TABLE 1:** Studies selected for combined analyses.

No.	Study	Sample	Target types	Observations
1	Torres‐Marín et al. (2017), experiments 1 and 2	40, 40	Face expressions	
2	Marotta et al. (2018)	35	Arrows vs. Eyes	
3	Marotta & Lupiáñez (2018)	17	Faces: Upright vs. Inverted	Inverted faces were removed
4	Marotta et al. (2019)	27	Arrows vs. Eyes	
5	Hemmerich et al. (2022), experiments 1 and 2	35, 33	Arrows vs. Eyes	
6	Narganes‐Pineda et al. (2022), experiment 1	24	Arrows vs. Eyes	
7	Marotta et al. (2022)	36	Face expressions	
8	Bonventre and Marotta (2023), experiments 1 and 2	24, 22	Arrows vs. Hand vs. Eyes	Hand targets were removed
9	Ishikawa et al. (2024), experiments 1 and 3	70, 54	Arrows vs. Cat face/robot face vs. Eyes	Cat face and robot face targets were removed
10	Tanaka, Ishikawa, et al. (2023), experiments 1A and 1B	38, 19	Faces: Upright vs. Inverted	Inverted faces were removed
11	Dalmaso et al. (2023)	191^a^	Arrows vs. Hand vs. Face	Hand targets were removed

*Note*: The samples are separated by a comma when the study has more than one experiment.

^a^ One participant was removed due to a higher rate of trials without response (≈64%).

It is important to note that several experiments, including those by Hemmerich et al. (2022) and Bonventre and Marotta (2023, experiment 2), presented target types within blocks. However, since this experimental design did not significantly improve the model fit (see Table S1), we excluded it as a relevant factor in the model formula.

### Analyses and design

The analyses were performed using RStudio (Posit team, 2024), and the script is available on the OSF webpage (see Data Availability Statement). We adopted the same cut‐off criterion for outliers and data pruning as in most original studies, removing reaction times shorter than 200 ms and longer than 1300 ms for simplicity and comparability, including Dalmaso et al. (2023) who originally used a 3 SD trimming procedure. Both correct and incorrect responses were considered, and the proportion of trials removed is reported in Table S2.

CAFs were computed by sorting reaction times from fastest to slowest before being divided into five 20% bins (e.g. Hübner & Töbel, 2019). Accuracy rates were then calculated for each participant within each condition (i.e. target type, congruency and bin) by experiment. We opted to conduct the analyses using 5 bins,^1^ although as it is typically expected to find modulations within the fastest responses, we performed the same contrast analyses for models MA and MB, which compared social and non‐social stimuli, using four 25% bins (see Appendix S1).

The datasets were combined to formulate three models (Table 2). Models MA and MB included all studies listed in Table 1, covering 16 experiments and 705 participants. We did not consider the emotional expression condition, collapsing the data across target type. In model MA, cropped eyes and faces were categorized as social stimuli, and arrows as non‐social stimuli, leading to a three‐way design: target type (social vs. non‐social), congruency (congruent vs. incongruent) and bin (5 levels). Model MB differentiated faces and cropped eyes resulting in a three‐way design: target type (face vs. eyes vs. arrows), congruency (congruent vs. incongruent) and bin (5 levels).

**TABLE 2:** Models formulated for CAF analyses.

Models	Studies	N° of datasets	Design
MA	All the studies listed in Table 1	16	Target type (social vs. non‐social), congruency (congruent vs. incongruent) and bin (5 levels)
MB	All the studies listed in Table 1	16	Target type (face vs. eyes vs. arrows), congruency (congruent vs. incongruent) and bin (5 levels)
MC	Torres‐Marín et al. (2017, experiments 1 and 2) and Marotta et al. (2022)	3	Expression (anger vs. fear vs. happiness vs. sadness vs. neutral), congruency (congruent vs. incongruent) and bin (5 levels)

*Note*: The combined dataset for models MA and MB was the same, except that in MA, we categorized both faces and eyes as social stimuli.

Model MA was based on previous research linking the RCE to eye‐gaze processing, regardless of using complete faces or cropped eyes. However, processing whole faces might add extra information. Model MB addresses this, with MA serving as a baseline for comparison. MB could offer new insights, as Hemmerich et al.'s experiment 3 (2022) is the only study directly comparing faces and eyes in this context. Additionally, considering faces as a complex background, this distinction may clarify whether the dissociation results from a background segregation effort.

Model MC aimed to explore differences related to emotional expression conditions at a distributional level by combining data from two studies (i.e. three experiments). The design included: expression (anger vs. fear vs. happiness vs. sadness vs neutral), congruency (congruent vs. incongruent) and bin (5 levels). Additionally, model MC will allow us to observe whether the patterns in model MB are stable using similar studies with face targets only.

Prior to modelling, k‐fold cross‐validation (Geisser, 1975) assessed dataset suitability for linear mixed modelling (LMM) or beta regression. LMM was chosen based on mean squared errors (MSEs). We used the lme4 (Bates et al., 2015) and lmerTest (Kuznetsova et al., 2017) R packages for fitting and testing. The best fit was determined through hierarchical comparisons, using the AIC and BIC criteria, along with the likelihood ratio test (*χ* ^2^). Subsequently, the best fitting models were identified (see Appendix S1), and significance of main effects and interactions was assessed through several analyses of variance (ANOVA).

For models MA and MB, the arrangement included fixed effects of target type, congruency, bin and their interactions. Given the complex structure of the combined datasets considered grouping and strategies to enhance model fitting (Barr, 2013; Barr et al., 2013; Brauer & Curtin, 2018). Participants were modelled as nested within studies using crossed effects syntax (see implicit nesting in Schielzeth & Nakagawa, 2013). Target type conditions were treated as study‐specific features, modelling inter‐study variations based on target type, while also acknowledging the specific structure within each study regarding congruency and bin factors. Regarding model MC, due to the similarities between the studies used, the model was simplified to include only a random intercept by participant. The rationale behind the grouping structures is described in Appendix S1.

The results presented here are from the best‐fitting models (Table 3), with a comprehensive account available in Appendix S1. Post‐hoc contrasts, where applicable, were performed using Tukey's HSD test within the mixed‐effects models framework, via the emmeans package in R (Lenth, 2023). Reported values represent the estimated marginal means for accuracy (EM Acc), reflecting the model structure. Contrast analyses employed 95% confidence intervals (CIs) and the Kenward‐Roger method (1997) for estimating the degrees of freedom. Partial eta squared ($\eta_{p}^{2}$) was estimated using the effectsize R package (Ben‐Shachar et al., 2020). Effect sizes (*d*) for post‐hoc contrasts were calculated following Westfall et al. (2014; see also Brysbaert & Stevens, 2018) using the EM Acc.

**TABLE 3:** Best‐fitting models.

Model	Target type condition	Formula
MA	Social vs. Non‐social	Acc ~ TargetType * Congruency * Bin + (1|Study:id) + (1|Study:TargetType) + (1|Study:TargetType:Congruency:Bin)
MB	Face vs. Eyes vs. arrows	
MC	Emotional expressions (anger, fear, happy, neutral and sad)	Acc ~ Expression * Congruency + Congruency * Bin + (1|Study:id)

## RESULTS AND DISCUSSION

The models with the best fit are outlined in Table 3, models MA and MB allowed the three‐way interaction, whereas model MC allowed the two‐way interactions, between expression and congruency, and between congruency and bin.

### Model MA

**FIGURE 1:**
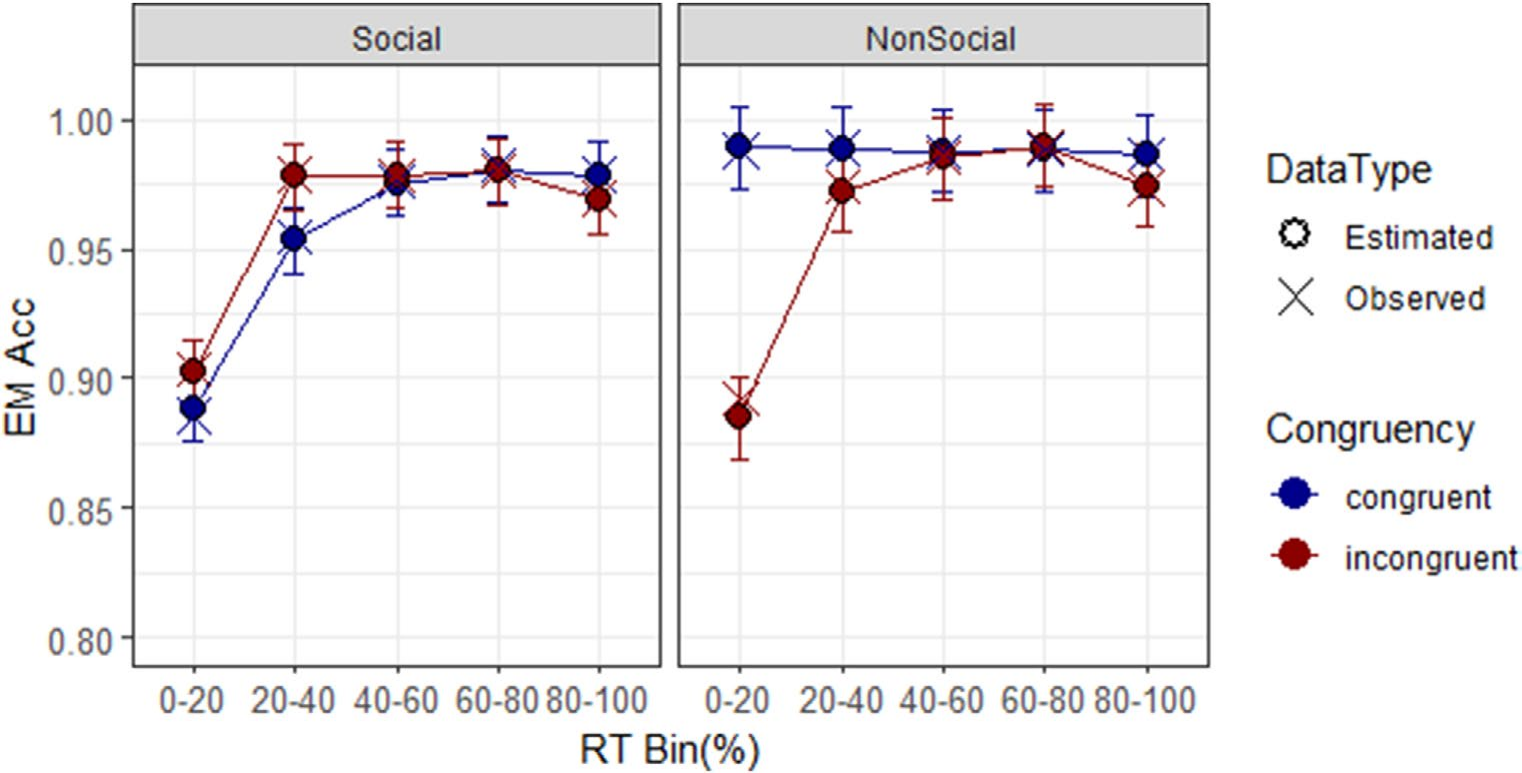
CAF for model MA. The *y*‐axis represents the estimated marginal means of accuracy rate (EM Acc). The bars represent the 95% confidence intervals of the EM Acc. Observed values are represented by ‘×’ and estimated values by ‘○’.

The analysis of variance (ANOVA) on model MA (Figure 1) indicated a significant main effect of target type, *F*(1, 22.491) = 7.06, *p* = .0142, $\eta_{p}^{2}$ = .239, with social stimuli associated with lower accuracy (0.958, SE = 0.004) than non‐social stimuli (0.975, SE = 0.005). Congruency was also significant, *F*(1, 223.383) = 14.81, *p* = .0002, $\eta_{p}^{2}$ = .062, with congruent trials being more accurate (0.972, SE = 0.004) than incongruent trials (0.961, SE = 0.004). Bin main effect was significant too, *F*(4, 223.383) = 91.04, *p* < .0001, $\eta_{p}^{2}$ = .620, indicating lower accuracy rates in bin 1 compared to subsequent bins.

The interaction between target type and congruency was significant, *F*(1, 223.383) = 38.08, *p* < .0001, $\eta_{p}^{2}$ = .146. In congruent conditions, participants were less accurate with social stimuli (Acc_soc_ = 0.955, SE = 0.004) than with non‐social (Acc_non‐soc_ = 0.988, SE = 0.005), *t*(28.8) = −4.90, *p* < .0001, *d* = −.344. In the incongruent condition no significant differences were observed (*p* = .974). Target type and bin interacted as well, *F*(4, 223.383) = 5.84, *p* = .0002, $\eta_{p}^{2}$ = .095, with social targets, in bin 1, being less accurate than non‐social targets (Acc_soc_ = 0.896, SE = 0.005 vs. Acc_non‐soc_ = 0.937, SE = 0.006), *t*(27.1) = −5.11, *p* < .0001, *d* = −.435. The interaction between congruency and bin was also significant, *F*(4, 223.383) = 11.68, *p* < .0001, $\eta_{p}^{2}$ = .173, in bin 1, it was observed higher accuracy rate with congruent (Acc_cong_ = 0.939, SE = 0.005) than incongruent trials (Acc_inc_ = 0.894, SE = 0.005), *t*(196) = 7.60, *p* < .0001, *d* = .477.

Crucially, the three‐way interaction was significant, *F*(4, 223.383) = 18.29, *p* < .0001, $\eta_{p}^{2}$ = .247. Post‐hoc contrasts (Table S5), revealed significant differences for non‐social targets in bin 1, obtaining higher accuracy rate in congruent (Acc_cong_ = 0.989, SE = 0.008) than incongruent trials (Acc_inc_ = 0.885, SE = 0.008), *t*(188) = 11.28, *p* < .0001, *d* = 1.10. In contrast, in bin 2, social targets showed higher accuracy in incongruent (Acc_inc_ = 0.871, SE = 0.006) than congruent trials (Acc_cong_ = 0.994, SE = 0.006), *t*(208) = −7.27, *p* < .0001, *d* = −.259. Furthermore, comparisons between bins (Table S6), revealed that social stimuli, at bin 1, exhibited lower accuracy rates than subsequent Bins in both, congruent (*t* = −12.29 to −8.65, *p* < .0001, *d* = −.944 to −.682) and incongruent conditions (*t* = −10.29 to −8.83, *p* < .0001, *d* = −.811 to −.696), whereas non‐social targets, showed the same pattern only in incongruent trials (*t* = −11.34 to −9.45, *p* < .0001, *d* = −1.11 to −.922). Additionally, social stimuli, in the congruent condition, also showed lower accuracy in bin 2 compared to bins 3–5 (*t* = −3.64 to −2.96, *p* < .0500, *d* = −.287 to −.233).

Interestingly, as typically observed, modulations were present within the fastest responses of the distribution. With non‐social targets, incongruent trials exhibited lower accuracy in the first bin compared to subsequent bins, while accuracy rates remained stable in congruent trials. Additionally, accuracy was significantly higher in congruent than in incongruent trials in the first bin. This common pattern in conflict inhibition research (e.g. Torres‐Quesada et al., 2022; Ulrich et al., 2015; van Campen et al., 2017; van Maanen et al., 2019) suggests that the initial boost of irrelevant spatial information, overseen by the automatic mechanism, increased the probability of selecting incorrect responses in incongruent trials (automatic response capture; Torres‐Quesada et al., 2022). From the second bin onwards, the automatic mechanism is reduced, leading to increased accuracy in both congruency conditions, as predicted by different models (Hommel, 1993; Ridderinkhof, 2002a, 2002b; Ulrich et al., 2015).

However, with social stimuli, the patterns observed in incongruent trials across bins were also observed in congruent conditions. Moreover, not only that, but congruent trials resulted in even worse performance than incongruent trials within the fastest responses, with significant differences in bin two. The 4‐bin model confirmed this pattern, highlighting modulations within the fastest responses (see Appendix S1). From a temporal dynamic perspective, this dissociation occurs at earlier processing stages, when task‐irrelevant information is still being processed. However, the automatic activation of the response to the irrelevant location would not lead to errors in congruent trials. This implies that additional information within the automatic processing, besides spatial location, interferes with typical patterns, ultimately modulating the congruency effect in terms of accuracy.

### Model MB

**FIGURE 2:**
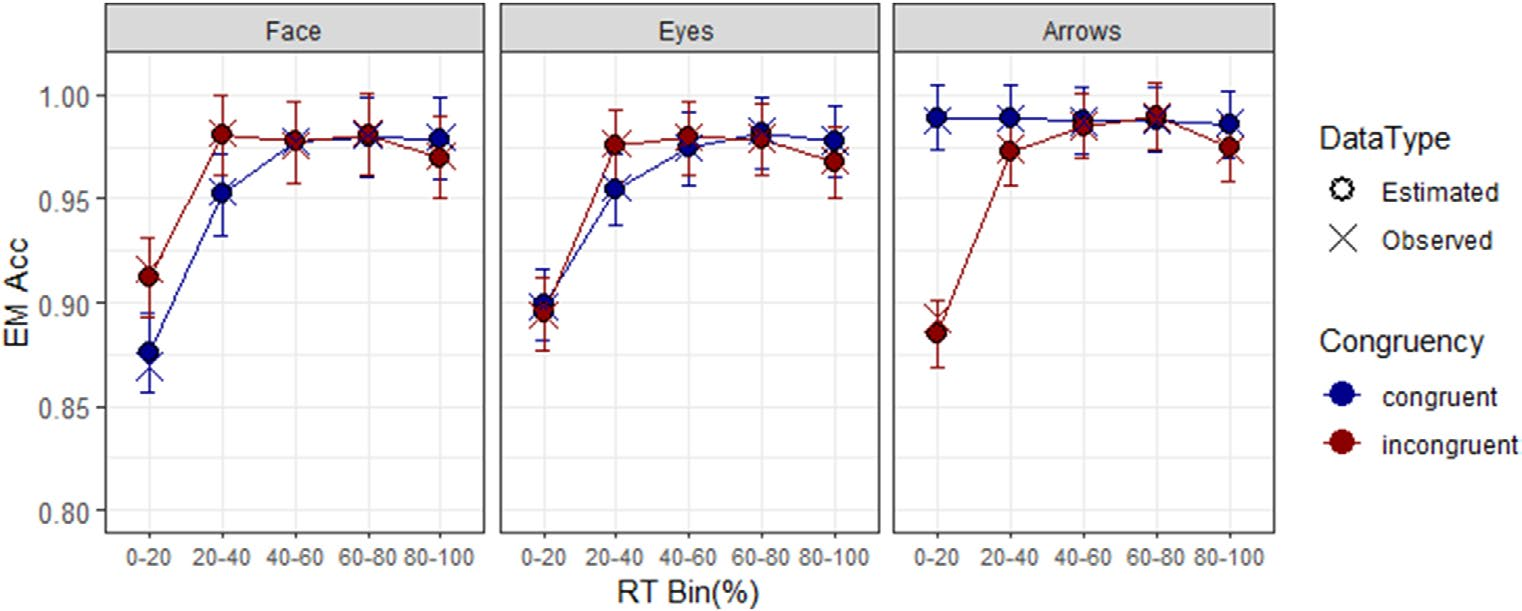
CAF for model MB. The *y*‐axis represents the estimated marginal means of accuracy rate (EM Acc). The bars represent the 95% confidence intervals of the EM Acc. Observed values are represented by ‘×’ and estimated values by ‘○’.

For model MB (Figure 2), the analyses of variance revealed a marginal effect of target type *F*(2, 23.38) = 3.40, *p* = .0507, $\eta_{p}^{2}$ = .225, with arrows (0.975, SE = 0.005) showing higher accuracy rate than face (0.959, SE = 0.006) and eyes targets (0.958, SE = 0.005). The main effect of bin was significant, *F*(4, 230.57) = 106.52, *p* < .0001, $\eta_{p}^{2}$ = .649, with lower accuracy rates in bin 1 compared to the subsequent bins and, lower accuracy rate in bin 2 than 4.

The interaction between target type and congruency was significant, *F*(2, 230.91) = 19.47, *p* < .0001, $\eta_{p}^{2}$ = .144. In the congruent condition, arrows (Acc_arrows_ = 0.988, SE = 0.006) showed higher accuracy than face (Acc_face_ = 0.953, SE = 0.007), *t*(31.3) = −4.08, *p* < .0010, *d* = −.369, and eyes targets (Acc_eyes_ = 0.957, SE = 0.006), *t*(27.1) = −3.98, *p* = .0013, *d* = −.322. No differences were observed between face and eyes targets, and no differences in the incongruent condition. The interaction between target type and bin was also significant, *F*(8, 230.91) = 2.89, *p* = .0043, $\eta_{p}^{2}$ = .091. In bin 1, both social stimuli, face (Acc_face_ = 0.894, SE = 0.008) and eyes (Acc_eyes_ = 0.897, SE = 0.007) showed lower accuracy rates than arrows (Acc_non‐soc_ = 0.937, SE = 0.008), *t*(61.4) = −4.17, *p* < .0010, *d* = .452 & *t*(57.1) = −4.30, *p* < .001, *d* = .424, respectively. Additionally, congruency and bin interacted as well, *F*(4, 230.57) = 4.96, *p* = .0007, $\eta_{p}^{2}$ = .079, in bin 1 congruent trials (Acc_cong_ = 0.921, SE = 0.005) exhibited higher accuracy rates than incongruent trials (Acc_inc_ = 0.897, SE = 0.005), *t*(190) = 4.04, *p* < .0001, *d* = .252.

Importantly, the three‐way interaction was again significant, *F*(8, 230.91) = 9.56, *p* < .0001, $\eta_{p}^{2}$ = .249. The contrasts analyses (Table S5), in bin 1 with face targets, revealed lower accuracy rate in congruent (Acc_cong_ = 0.876, SE = 0.010) than incongruent trials (Acc_inc_ = 0.912, SE = 0.010), *t*(178) = −3.24, *p* = .0014, *d* = −.385. Conversely, arrow targets exhibited higher accuracy rates in congruent (Acc_cong_ = 0.989, SE = 0.008) than incongruent trials (Acc_inc_ = 0.885, SE = 0.008), *t*(180) = 11.08, *p* < .0001, *d* = 1.10. In bin 2, both, face (Acc_cong_ = 0.952, SE = 0.010 vs. Acc_inc_ = 0.981, SE = 0.010) and eyes targets (Acc_cong_ = 0.954, SE = 0.009 vs. Acc_inc_ = 0.976, SE = 0.009) showed lower accuracy rates in congruent compared to incongruent trials, *t*(178) = −2.54, *p* = .0120, *d* = −.302, and *t*(218) = −2.07, *p* = .04, *d* = −.223, respectively. No differences were observed in the other bins.

On the other hand, the contrasts between bins across congruency levels (Table S6), revealed that both face and eyes stimuli, at bin 1, exhibited lower accuracy rates than subsequent bins in both congruent (*t*_face_ = −9.18 to −6.74; *p* < .0001, *d* = −1.09 to −.802; *t*_eyes_ = −8.07 to −5.43; *p* < .0001, *d* = −.869 to −.584) and incongruent conditions (*t*_face_ = −6.07 to −5.08; *p* < .0001, *d* = −.722 to −.605; *t*_eyes_ = −11.48 to −9.90; *p* < .0001, *d* = −.890 to −.769), whereas arrows target, showed lower accuracy only in incongruent trials in bin 1 than the other bins (*t*_arrows_ = −8.26 to −7.14; *p* < .0001, *d* = −1.10 to −.919).

In model MB, the same patterns were observed as in model MA with non‐social stimuli. However, when separating the social stimuli into faces and eyes, the reversion of the congruency effect affected faces earlier, showing reduced accuracy in congruent trials in bins 1 and 2 compared to incongruent trials, while with eyes, this dissociation was observed only in bin 2. This pattern with faces was also observed in the 4‐bin model. However, with eyes, although the direction was the same as in the 5‐bin model, the dissociation was not significant (see Appendix S1). Additionally, for social stimuli, performance was lower in the first bin compared to the rest of the bins in both congruency conditions in the 4‐bin and 5‐bin models.

Similarly, the modulations occurred within the fastest responses, indicating that additional information beyond spatial location interfered with the usual patterns during early stages of automatic processing. On one hand, it is possible to suggest that faces provide additional social information that facilitates the dissociation from earlier stages, increasing the strength and duration of the effect. This distinction highlights the quick activation of automatic information for faces (other than location), possibly attributed to their direct recognition (Freiwald et al., 2016; Nemrodov et al., 2014; Watanabe et al., 1999). Conversely, with cropped eyes, this activation might be slightly delayed or reduced, as processing isolated eye stimuli is less familiar. If this is the case, 4‐bin model might hide some relevant information since it mixes information between faster and middle responses latencies, giving the same pattern observed as 5‐bin model, but without reaching significance (Figure S2).

Moreover, considering the temporal sequence of events observed in Marotta et al. (2019), the P1 component exhibited a main effect of congruency without interacting with target type. This suggests that modulations of sensory processing, attributed to attentional orienting (Luck, 1995), may be due to the similar handling of spatially irrelevant information across both stimulus types. Conversely, the subsequent N170 component activation demonstrated a significant difference between eye‐gaze and arrow stimuli. Although this early dissociation was not observed in terms of congruency, it might indicate an initial distinction between socially relevant stimuli from non‐social stimuli (Bentin et al., 1996; Sagiv & Bentin, 2001). This early distinction could influence subsequent processing stages by introducing new information related to the social nature of the stimuli, such as the *looking vector* dimension (Hemmerich et al., 2022), described as a set of complex social interactions, including gaze direction processing.

On the other hand, based on non‐social accounts (Chen et al., 2022; Tanaka et al., 2024; Tanaka, Oyama, et al., 2023), perceptual factors might modulate the observed patterns. However, perceptual influences alone, without considering eye‐gaze, are unlikely to improve performance in incongruent trials compared to congruent ones. This should impact both congruency levels equally, reducing accuracy without showing a reversion.

Alternatively, the dual‐stage hypothesis (Tanaka et al., 2024; Tanaka, Oyama, et al., 2023) suggests the RCE is produced during later processing stages, after segregating directional information from a complex background. According to the activation–suppression hypothesis, the inhibitory process gradually builds up (Ridderinkhof, 2002a, 2002b), making it more efficient for slower responses (e.g. van Campen et al., 2017). Thus, reversion in accuracy should occur during middle or slower responses and later with faces due to their perceptual complexity. Conversely, if active suppression starts earlier than assumed (Tanaka, Oyama, et al., 2023), it could modulate performance by interacting with early social information processing.

### Model MC

**FIGURE 3:**
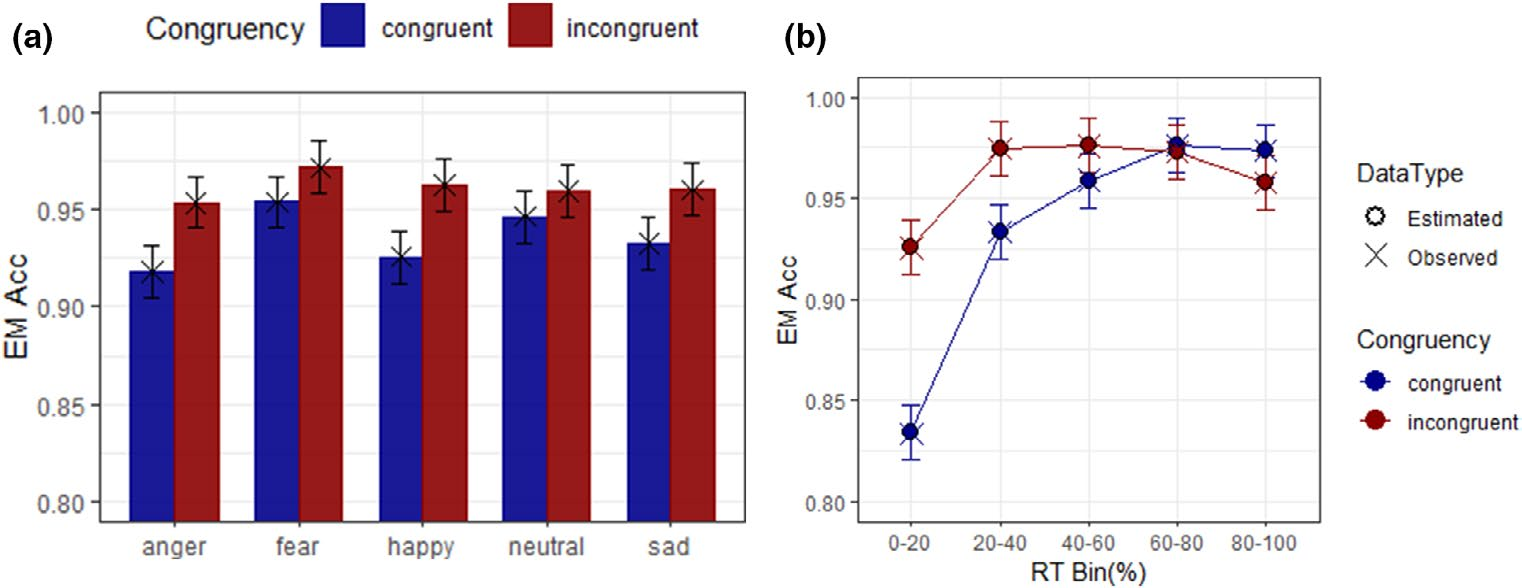
Interactions figures allowed for model MC. (a) Bar plot of congruency by expression; (b) CAF plot of congruency by bin. The *y*‐axis represents the estimated marginal accuracy rates (EM Acc), the bars represent the 95% confidence interval of the EM Acc. Observed values are represented by ‘×’ and estimated values by ‘○’.

The ANOVA on model MC (Figure 3), revealed a main effect of Expression, *F*(4, 5667) = 11.26, *p* < .0001, $\eta_{p}^{2}$ = .008. Anger condition (0.936, SE = 0.006) showed lower accuracy rate than fear (0.963, SE = 0.006), *t*(5667) = −6.34, *p* < .0001, *d* = −.229, and neutral faces (0.953, SE = 0.006), *t*(5667) = −4.01, *p* = .0006, *d* = −.145. With fear faces, the accuracy was higher than with happy (0.944, SE = 0.006), *t*(5667) = 4.36, *p* = .0001, *d* = .158, and sad (0.946, SE = 0.006) faces, *t*(5667) = 3.89, *p* = 0.0010, *d* = .141. Congruency showed a significant effect, *F*(1, 5667) = 97.06, *p* < .0001, $\eta_{p}^{2}$ = .017, with incongruent trials (0.961, SE = 0.006) being more accurate than congruent trials (0.935, SE = 0.006). The main effect of bin was also significant, *F*(4, 5667) = 171.280, *p* < .0001, $\eta_{p}^{2}$ = .108. The patterns were similar for social targets in previous models, being bin 1 less accurate than the subsequent bins. Additionally, it was observed that bin 2 was significantly less accurate than bins 3 and 4.

Model MC showed as significant the two‐way interactions, between expression and congruency, *F*(4, 5667) = 3.21, *p* = .0121, $\eta_{p}^{2}$ = .002, and between congruency and bin, *F*(4, 5667) = 50.69, *p* < .0001, $\eta_{p}^{2}$ = .035. In the first case, the contrasts analyses between expressions showed that in the congruent condition, anger (0.918, SE = 0.007) were less accurate than fearful expression (0.954, SE = 0.007), *t*(5667) = −5.99, *p* < .0001, *d* = −.306, and neutral (0.946, SE = 0.007), *t*(5667) = −4.73, *p* < .0001, *d* = .242. In the same direction, happy (0.926, SE = 0.006) showed lower accuracy than neutral, *t*(5667) = −3.46, *p* = .0050, *d* = −.177. Conversely, fear was more accurate than happy, *t*(5667) = 4.72, *p* < .0001, *d* = .241, and sad faces (0.932, SE = 0.007), *t*(5667) = 3.60, *p* = .0029, *d* = .184. In the incongruent condition, only anger (0.954, SE = 0.007) was less accurate than fear (0.971, SE = 0.007), *t*(5667) = −2.98, *p* = .0239, *d* = −.153. In addition, when comparing the congruency levels by expression, in all the cases, the accuracy rate was significantly higher for incongruent than congruent trials (*t* = −6.23 to −2.19, *p* < .05, *d* = −.319 to −.112).

With respect to the interaction between congruency and bin, the contrasts across bins, revealed significantly lower accuracy rates with congruent than incongruent trials in bin 1 (Acc_cong_ = 0.834, SE = 0.007 vs. Acc_inc_ = 0.926, SE = 0.007), *t*(5667) = −15.35, *p* < .0001, *d* = −.785; bin 2 (Acc_cong_ = 0.933, SE = 0.007 vs. Acc_inc_ = 0.975, SE = 0.007), *t*(5667) = −6.96, *p* < .0001, *d* = −.356; and bin 3 (Acc_cong_ = 0.959, SE = 0.007 vs. Acc_inc_ = 0.976, SE = 0.007), *t*(5667) = −2.90, *p* = .0038, *d* = −.148. In contrast, in bin 5, a significant higher accuracy rates in congruent than incongruent trials was observed (Acc_cong_ = 0.973, SE = 0.007 vs. Acc_inc_ = 0.958, SE = 0.007), *t*(5667) = 2.61, *p* = .0091, *d* = .134.

Similarly, the comparisons between bins by congruency revealed similar patterns to those observed in the previous models with social targets. In the congruent condition, the accuracy was significantly lower in bin 1 than the subsequent bins (*t* = −23.87 to −16.67, *p* < .0001, *d* = −1.22 to −.852). In addition, in model MC, it was observed also significant differences between bin 2 and the subsequent bins (*t* = −7.21 to −4.27, *p* < .0001, *d* = −.369 to −.218), and between bins 3 and 4, *t*(5667) = −2.94, *p* = .0273, *d* = −.150. In the incongruent condition, it was observed lower accuracy rates in bin 1 than the subsequent bins (*t* = −8.48 to −5.41, *p* < .0001, *d* = −.433 to −.277). Conversely, it was observed lower accuracy rates in bin 5, when compared with bins 2, *t*(5667) = 2.86, *p* = .0346, *d* = .146 and 3, *t*(5667) = 3.069, *p* = .0184, *d* = .157.

Thus, model MC showed a stable pattern across the distribution within the CAF and the same reversion regarding accuracy for face stimuli as observed in model MB. Interestingly, the decrease in accuracy rates in congruent trials was observed from bin 1 to bin 3 compared to incongruent trials. Moreover, the comparison across bins showed a reduction in accuracy that lasted longer (i.e. bin 3 vs. bin 4). Again, these dissociations were observed within the fastest responses, which are related to earlier stages of processing. Importantly, this model chose methodologically similar studies, whose resemblance allowed a simpler modelling structure with less variance between studies.

Although model MC did not allow for the three‐way interaction, and therefore, we did not analyse the expressions' facilitation at a distributional level, the emotional expressions might have added some extra information. Indeed, congruent trials showed modulations that were not apparent in incongruent trials when comparing expressions (Table S9). Additionally, we observed a reduction in accuracy for congruent trials within the five emotional expressions, with the neutral expression showing the lowest t‐ratio (Table S7). These observations suggest that adding extra social information (i.e. emotional expressions) might modulate the patterns observed within the fastest responses in CAFs.

## GENERAL DISCUSSION

This study aimed to investigate the differences in attentional processing between social and non‐social stimuli using a distributional approach, particularly focusing on the Conditional Accuracy Function (CAF; Heitz, 2014; Wood & Jennings, 1976). Accuracy rates were divided into equally sized bins based on reaction time distribution. To achieve this, we combined the datasets from 11 studies (listed in Table 1) to fit three models to the data from 705 participants: MA compares social (eyes and faces) with non‐social (arrows) targets, MB compares faces, eyes and arrows, and MC assessed variations in accuracy rates based on emotional expressions within face targets.

With non‐social targets, our analyses highlighted modulations within the fastest responses, replicating previous results. In models MA and MB, incongruent trials exhibited lower accuracy rates in the first bin compared to subsequent bins, whereas accuracy rates remained stable throughout the distribution in congruent trials. Moreover, accuracy was significantly higher in congruent than in incongruent trials in the first bin. These findings extend previous research reporting more errors in incongruent trials with non‐social targets (Bonventre & Marotta, 2023, experiment 1; Ishikawa et al., 2021, experiment 1, 2024, experiment 3; Marotta et al., 2018, 2019), suggesting these differences are primarily evident within the fastest responses. These patterns are consistent with either a passive‐decay model (e.g. Hommel, 1993), or an active‐inhibition model (e.g. Ridderinkhof, 2002a, 2002b).

However, with social stimuli, the CAFs exhibited three main variations. First, both congruent and incongruent conditions showed reduced accuracy within the fastest responses. Second, congruent (incongruent) trials resulted in worse (better) performance than incongruent (congruent) trials in the initial bins. Third, faces elicited and enhanced these modulations earlier than eyes stimuli. These patterns do not align with the expected facilitation of correct response selection in congruent trials by the automatic mechanism's initial boost (Lu & Proctor, 1995; Torres‐Quesada et al., 2022; Ulrich et al., 2015), or the reduced performance in incongruent trials. Overall, these observations suggest that additional information influenced the temporal dynamics of conflict elicitation and resolution during the activation of the automatic mechanism.

The reduced performance for the fastest responses in both congruency levels in model MA could be explained by either social or non‐social accounts based on the difficulties in target processing (i.e. the target eyes are embedded and need to be extracted from the face). However, if task‐relevant information extraction were the sole cause, model MB should show larger differences in the main effects of target type or the 2 two‐way interactions (i.e. target type by congruency, and target type by bin), for faces and eyes, as faces are perceptually more complex and require more time to process. Alternatively, the distinction between socially relevant and non‐social stimuli might initially engage attentional resources similarly for social targets, with later differentiation between faces and eyes due to the interaction of perceptual complexity and social‐related processing mechanisms.

Regarding the initial distinction of social and non‐social stimuli, electrophysiological research has identified early neural indexes, particularly the N170 ERP component in scalp recordings and the N200 ERP component in intracranial recordings (Eimer, 2011; McCarthy et al., 1999; Rousselet et al., 2014). Faces and eyes typically elicit larger N170 (Rossion & Jacques, 2012), and N200 (Allison et al., 2002) components than other object types. In face detection tasks, heightened sensitivity to contralateral eye stimuli occurs between the P1 and N170 components, indicating this time window as critical for face processing (Rousselet et al., 2014; Schyns et al., 2003). Moreover, differences have been noted in processing faces, faces without eyes and isolated eyes with both the N170 (e.g. Bentin et al., 1996; Itier & Preston, 2018) and the N200 (e.g. Allison et al., 2002; McCarthy et al., 1999) components, suggesting different sources principally for ‘face‐N170’ and ‘eye‐N170’, which explains their distinct topographies and developmental courses (Itier et al., 2007; Itier & Preston, 2018; Miki et al., 2015; Nemrodov et al., 2014; Taylor et al., 2004).

After initial stimulus differentiation, interactions between social and non‐social information may emerge, influenced by perceptual configuration, task dynamics and the looking vector dimension. Evidence shows that the posterior superior temporal sulcus (pSTS), part of the social perception network (Yang et al., 2015), receives input from primary visual regions sensitive to social stimuli, such as eye‐gaze direction (Gobbini & Haxby, 2007; Hoffman & Haxby, 2000). Moreover, faces can be distinguished from other objects without conscious awareness (Morris et al., 2007), suggesting automatic processing. Gaze perception also triggers early, automatic attentional orienting (Brignani et al., 2009; Driver et al., 1999) in occipitoparietal regions (Ulloa et al., 2018). When the looking vector dimension integrates this social information into the automatic mechanism during social trials, it spills over into task‐relevant processing. The introduction of gaze direction information in incongruent trials would create a synergistic effect where automatic and controlled processes converge.

The eye‐contact hypothesis explains this effect: Participants implicitly perceive the eyes as looking at them in incongruent conditions, enhancing performance (Cañadas & Lupiáñez, 2012; Marotta et al., 2018). Initially, participants process spatially irrelevant information similarly for arrows and gaze. However, in social trials, the nature of the stimuli interferes with spatial information, triggering an eye‐contact episode within the automatic mechanism. Simultaneously, the controlled process gathers evidence to guide the decision towards the correct response. In contrast, in congruent trials, no eye‐contact episode occurs, and the controlled mechanism does not fully override the automatic mechanism at earlier stages. Variability in the automatic mechanism's strength over time, influenced by cognitive control (Banich, 2019; Soutschek et al., 2013; Ulrich et al., 2015), explains the pattern in slower responses, where accuracy differences between congruent and incongruent trials diminish.

This dynamic could explain the findings of Narganes‐Pineda et al. (2022) in implicit tasks where no congruency effect was observed. The looking vector might require the strength of the controlled, task‐relevant process to trigger the reversion. Importantly, Narganes‐Pineda et al.'s implicit tasks used a Type 3 ensemble, producing a Simon effect. In their experiment 3A (explicit task), participants responded orally to gaze direction, creating no overlap between stimuli and response (i.e. Type 4 ensemble; Kornblum, 1992); yet, a RCE was still observed. This suggests that the RCE might not be entirely stimulus–response related.

Although further exploration is needed to understand how different dimensional overlaps influence the processing of both stimulus types, it is possible that the congruency effect observed in accuracy occurs at different processing levels compared to those facilitating the RCE observed with RT. Tanaka, Oyama, et al. (2023) found that arrows and eye‐gaze lead to negative‐going delta plots (for characterization of the delta functions, see Speckman et al., 2008), indicating inhibition mechanisms for arrows but the strengthening of the RCE for eye‐gaze during middle and slower correct responses, with the reversion observed from the fastest responses only for social stimuli. These differences between the CAFs and delta plots align with the multiple‐stage and multiple loci theories (Banich, 2019; De Houwer, 2003; Parris et al., 2022), suggesting that different processing levels contribute to the overall conflict effect (Viviani et al., 2024).

The idea that control mechanisms adapt to context or task‐related conflicts is not new (Funes et al., 2010; Schlaghecken & Martini, 2012; Soutschek et al., 2013). The Stroop effect, for instance, can be generated and inhibited at multiple levels, from stimulus presentation to response execution (Banich, 2019; Torres‐Quesada et al., 2022), demonstrating the adaptability of control mechanisms. This adaptability is crucial for complex stimuli like faces, where social information operates across various processes, from perception to higher order cognitive functions (Yang et al., 2015).

Investigating the potential influence of joint attention or joint distraction episodes could provide additional insights. Our primary focus was on automatic mechanisms, as CAF modulations typically occur within the fastest responses, associated with automatic processing. The eye‐contact hypothesis offers a plausible explanation for these patterns. Conversely, joint attention and distraction likely involve higher order cognitive processes (Baron‐Cohen, 1994; Hemmerich et al., 2022), which require more time to activate and may affect middle and slower responses modulations that CAFs might not capture.

While the looking vector dimension provides one possible explanation, other factors may also be at play. Testing this hypothesis with targets like tongues, which have been shown to produce a reversion (Tanaka et al., 2024), without triggering an eye‐contact episode, could be insightful, given that tongues might still convey social information. The mouth region is important in face processing (e.g. Itier & Preston, 2018; Luo et al., 2013), and the tongue has been shown to elicit a cueing effect similar to gaze (e.g. Downing et al., 2004, experiment 1) and plays a role in emotional expression (e.g. Rozin et al., 1994). Therefore, future research should explore alternative explanations by considering perceptual factors and temporal task dynamics, as suggested by non‐social theories.

Finally, it is important to acknowledge the study's limitations. The social version of the spatial Stroop task results in minimal errors, which may limit the generalizability of comparisons. Nuanced differences between models MA and MB could arise from study variations, as speed‐accuracy trade‐off can influence outcomes, particularly conditions emphasizing speed tends to amplify CAF effects in incongruent trials (e.g. Riesel et al., 2019; Wylie et al., 2009). Partitioning the RT distribution into bins has faced criticism for temporal reduction and arbitrary bin selection (van Leeuwen et al., 2019; van Maanen et al., 2019). However, this method was specifically designed to analyse accuracy relative to response latencies, considering their distributional characteristics (Luce, 1986; Pachella, 1973; Wood & Jennings, 1976). Given our aims, and the consistent modulations observed within the fastest responses, regardless of bin number (e.g. Hübner & Töbel, 2019; Ulrich et al., 2015; Wolkersdorfer et al., 2020), we found this approach appropriate. Similar modulations have been noted in studies with neurostimulation techniques and diverse populations (e.g. Ambrosi et al., 2020; van Campen et al., 2017; van Wouwe et al., 2016).

For a more detailed pattern exploration and hypothesis testing, it is recommended to complement this procedure with others, such as averaging parameter approaches (Rouder & Speckman, 2004). This can be achieved by modelling data using the Drift Diffusion Model (DDM; Ratcliff, 1978; see Myers et al., 2022) or the diffusion model for conflict tasks (DMC; see Ulrich et al., 2015). Future studies should use this approach.

## CONCLUSION

In conclusion, in this study an important dissociation has been observed in attentional processing between social and non‐social stimuli, through the use of a distributional approach such as that of the Conditional Accuracy Function (CAF). In particular, the evaluation of accuracy rates across different reaction time bins suggests the existence of relevant differences in response patterns, especially in the early bins. For non‐social stimuli such as arrows, congruent trials showed greater accuracy than incongruent ones, for which reduced accuracy was observed on fastest responses, thus confirming the hypothesis that the presence of relevant and irrelevant information generates a conflict that negatively impacts initial performance. On the contrary, for social stimuli, such as faces and eyes, an inverse pattern is observed with congruent trials being less accurate than incongruent ones in the first bins. This dissociation suggests that with social stimuli, not only the automatic capture by the irrelevant target location, but also other automatic processes might modulate decisions in unique ways, perhaps social, unanticipated by traditional models of conflict processing, which would indicate the adaptability of automatic mechanisms to manage the complexity of social information from the initial stages of processing. These findings help to understand how attention and spatial interference mechanisms differ depending on the nature of the stimuli and also highlight the importance of considering the temporal dynamics of attentional processing in future studies.

## AUTHOR CONTRIBUTIONS

**Renato Ponce:** Conceptualization; data curation; formal analysis; methodology; software; visualization; writing – original draft; writing – review and editing. **Juan Lupiáñez:** Conceptualization; data curation; funding acquisition; methodology; project administration; supervision; validation; writing – review and editing. **Carlos González‐García:** Data curation; methodology; validation. **Maria Casagrande:** Funding acquisition; supervision. **Andrea Marotta:** Conceptualization; data curation; funding acquisition; methodology; project administration; supervision; validation; writing – review and editing.

## CONFLICT OF INTEREST STATEMENT

None of the authors have any financial interest or conflict of interest.

## Supporting information


Appendix S1.

## Data Availability

The data, the supplemental material and the script of the analysis that support the findings of this study are openly available in OSF at https://osf.io/twbs3/.

## Footnotes

^1^
In studies utilizing a distributional approach, the number of bins chosen ranges from 3 to 10 (e.g. Ambrosi et al., 2020; Hübner & Töbel, 2019; Mittelstädt et al., 2022; van Maanen et al., 2019).
