# Supplementary material for: Exploring the spatial interference effects elicited by social and non‐social targets: A conditional accuracy function approach
Source: Br J Psychol. 2024 Sep 13;116(1):69–88. doi: 10.1111/bjop.12735 (PMC11724688; doi:10.1111/bjop.12735)
Supplement: Supplementary file 1 — Appendix S1. [file BJOP-116-69-s001.docx]

**Supplemental Material**

*Table of contents*

[*Impact of target presentation design on model fitting* 1](#_Toc172550124)

[*Trimming procedure* 2](#_Toc172550125)

[*Linear-Mixed model and beta regression comparisons* 3](#_Toc172550126)

[*Hierarchical comparison against the Null Model* 3](#_Toc172550127)

[*Contrast analyses models MA and MB* 5](#_Toc172550128)

[*Contrast analyses model MC* 8](#_Toc172550129)

[*Four-Bin models: MA and MB* 10](#_Toc172550130)

# *Impact of target presentation design on model fitting*

To assess the impact of target presentation method (i.e., within-block vs. between-blocks), we combined data from the studies by Hemmerich et al. (2022, experiments 1 & 2) and Bonventre & Marotta (2023, experiment 2), which comprise 3 experiments using within-block target presentations. In contrast, the between-block design was used in the studies by Marotta et al. (2018, 2019), and Bonventre & Marotta (2023, experiment 1), totaling 3 datasets. All these studies conducted arrow vs. eye-gaze comparisons, ensuring methodological consistency.

Linear mixed models were employed to contrast the saturated and full models against the null model. Both the saturated and full models included target type, congruency, bin, and their interactions as fixed effects, with participants nested within studies as random effects. The saturated model included the block design condition (within vs. between) as an additional predictor: *Acc ~ TargetType * Congruency * Bin + BlockDesign + (1|Study:id)*. The results, presented in Table 1, revealed that incorporating the ‘BlockDesign’ predictor did not significantly improve the fit.

| **Table 1.** Target type block presentation design analysis | | | | | |
| --- | --- | --- | --- | --- | --- |
| **Model** | **AIC** | **BIC** | ***χ²*** | **Df.** | **p-value** |
| Saturated | -8 451 | -8 310 | 0.75 | 1 | 0.388 |
| Full | -8 453 | -8 317 | 642.84 | 19 | < .0001 |
| Null | -7 848 | -7 829 | - | - | - |
| ***Note.*** The comparisons were tested using the likelihood ratio test, in a sequential manner, Saturated vs. Full, and Full vs. Null models. | | | | | |

# *Trimming procedure*

For the trimming procedure, we followed the same cutoff criterion used in most original studies, excluding reaction times shorter than 200ms and longer than 1300ms (Table 2). This method was applied across all studies, including Dalmaso et al. (2023), even though the original study utilized a trimming procedure based on 3 standard deviations (SDs). Additionally, our trimming procedure accounted for both correct and incorrect responses.

| **Table 2.** Error rates, remaining trials and trials removed by study | | | |
| --- | --- | --- | --- |
| **Study** | **Incorrect trials (%)** | **Remaining trials (%)** | **Trials removed (%)** |
| Torres-Marín et al. (2017), experiments 1 & 2 | 5.44%; 4.95% | 98.10%; 98.95% | .01% (*<200*), 1.15% (*zero*), .74% (*>1300*);  .01% (*<200*), 1.12% (*zero*), .92% (*>1300*) |
| Marotta et al. (2018) | 3.15% | 99.65% | .01% (*<200*), .06% (*zero*), .28% (*>1300*) |
| Marotta (Unpublished) | 2.03% | 99.49% | .00% (*<200*), .14% (*zero*), .37% (*>1300*) |
| Marotta et al. (2019) | 3.21% | 99.32% | .00% (*<200*), .30% (*zero*), .38% (*>1300*) |
| Hemmerich et al. (2022), experiments 1 & 2 | 2.23%; 4.45% | 99.14%; 98.88% | .00% (*<200*), .20% (*zero*), .66% (*>1300*);  .00% (*<200*), .53% (*zero*), .59% (*>1300*) |
| Narganes-Pineda et al. (2022), experiment 1 | 2.66% | 99.17% | .02% (*<200*), .31% (*zero*), .50% (*>1300*) |
| Marotta et al. (2022) | 5.37% | 99.00% | .03% (*<200*), .16% (*zero*), .81% (*>1300*) |
| Bonventre & Marotta (2023), experiments 1 & 2 | 2.25%; 3.08% | 99.61%; 99.37% | .00% (*<200*), .00% (*zero*), .39% (*>1300*);  .00% (*<200*), .06% (*zero*), .57% (*>1300*) |
| Ishikawa et al. (2022), experiments 1 & 3 | 3.99%; 4.14% | 99.13%; 99.36% | .11% (*<200*), .00% (*zero*), .76% (*>1300*);  .32% (*<200*), .00% (*zero*), .32% (*>1300*) |
| Tanaka et al. (2023a), experiments 1A & 1B | 5.13%; 4.20% | 99.20%; 99.84% | .08% (*<200*), .16% (*zero*), .56% (*>1300*);  .00% (*<200*), .08% (*zero*), .08% (*>1300*) |
| Dalmaso et al. (2023) | 2.69% | 99.71% | .01% (*<200*), .19% (*zero*), .09% (*>1300*) |
| ***Note.*** The reported error rates were calculated after the trimming procedure. The reported values are separated by a semicolon for studies with more than one experiment. The column ‘Remaining trials’ represents the percentage of correct and incorrect responses after the trimming procedure. The column ‘Trials removed’ represents the percentage of trials faster than 200ms (*<200*), without response (*zero)*, and slower than 1300ms (*>1300*). | | | |

The results showed that incorrect trials after trimming ranged from 2.03% to 5.44%. The remaining trials, both correct and incorrect, ranged from 98.10% to 99.85%. This aligns with the recommendations of Ülrich and Miller (1994), which suggest removing less than 0.5% of the data. We also reported separately trials without response (0.00% to 1.15%) and those faster than 200ms (0.00% to 0.11%). Trials slower than 1300ms ranged from 0.08% to 0.92%. Importantly, 7 datasets did not have trials under 200ms, 5 datasets had less than 0.05%, and the remaining datasets showed no trials under 200ms over 0.11%, minimizing the risk of losing important information since typical modulations in CAF literature are within the fastest responses (e.g., Amborsi et al., 2019; Ülrich et al., 2015). Consequently, the data removal was minimal, as recommended (e.g., Ratcliff, 1993; Ülrich & Miller, 1994).

In addition, the same analyses were performed by removing only trials without response. Models MA and MB showed consistent patterns throughout the distribution. Model MC displayed similar patterns, although the contrast between bin 3 and bin 4 in congruent trials was not significant. This change does not affect our conclusions. Although these results are not reported, the untrimmed datasets and the script are available on the OSF webpage (see data availability statement).

# *Linear-Mixed model and beta regression comparisons*

We evaluated linear mixed modeling (LMM) against beta regression (Ferrari & Cribari-Neto, 2004) using the k-fold cross-validation method (Geisser, 1975), employing 10 folds. We compared the mean squared errors (MSEs) across both methods for each full model. For LMM, we utilized the lme4 package, and for beta regression, the glmmTMB package (Bates et al., 2015; Brooks et al., 2017, respectively). In all three comparisons, LMM exhibited lower MSE values: *MSE-MA_LMM_ = .008093 vs MSE-MA_beta_ = .009515; MSE-MB_LMM_ = .008094 vs MSE-MB_beta_ = .009518; MSE-MC_LMM_ = .01229 vs MSE-MC_beta_ = .01061*

# *Hierarchical comparison against the Null Model*

To identify the best-fitting models and their closest competitors for further comparisons, we employed the likelihood ratio test along with the AIC and BIC criteria. We systematically assessed the models, starting from the most complex (the Full model) and progressing to the simplest (the null model), in a sequential manner (Table 3): Full versus Level1, followed by Level1 versus Level2, and so forth. The criterion for simplifying the Full model was based on the interaction among the three factors (i.e., target type, congruency, bin); thus, the Full model accounted for the three-way interaction. The Level1 model considered the two-way interactions between target type and congruency, and between congruency and bin, while the Level2 model included only the interaction between target type and congruency. Had any model not achieved a significant result, we would have added more levels, but this was not necessary.

Additionally, we evaluated various random parameters, considering the inherent grouping structure and strategies for improving model fit (Barr, 2013; Barr et al., 2013; Meteyard & Davies, 2020). Participants, the basic unit of analysis, were modeled as nested within studies. The R syntaxis for nested designs is ‘*(1|Study/id)*’ or its alternative ‘*(1|Study) + (1|Study:id)*’, as described in Bates et al. (2015, Table 2). However, the variance for the inclusion of intercept by Study -i.e., *(1|Study)*- was zero, producing a singular fit. Therefore, we used a crossed effects syntax as suggested in Schielzeth & Nakagawa (2013): “Nested effects can usually be fitted using the syntax for crossed effects if the coding reflects implicit nesting” (p. 21).

The same implicit nesting considerations were applied to the other random parameters. Target type conditions were treated as unique to each study, allowing for modeling variations between studies based on target type -i.e., *(1|Study:TargetType*)-, while also considering each study’s unique structure regarding congruency and bin factors -*i.e., (Study:TargetType:Congruency:Bin)*-. Similarly, for model MC, introducing a random intercept for each study led to zero variance. In addition, the studies included were methodologically similar, prompting a simplification of the model to include only a random intercept for participants within each study.

| **Table 3.** Hierarchical comparison levels within each model. | | |
| --- | --- | --- |
| **Levels** | **Fixed** | **Random** |
| Full | *Acc ~ TargetType * Congruency * Bin* | *+ (1\|Study:id) + (1\|Study:TargetType) + (1\|Study:TargetType:Congruency:Bin)*  *MC: + (1\|Study:id)* |
| Level1 | *Acc ~TargetType * Congruency + Congruency * Bin* |  |
| Level2 | *Acc ~ TargetType * Congruency* |  |
| Null | *Acc ~ 1* |  |
| ***Note.*** For model MC, the predictor ‘TargetType’ was replaced by ‘Expression’. | | |

The results of the hierarchical comparison are described in Table 4. For model MA, the comparison among the Full (best fit) and Level1 models (second-best fit) revealed a significant improvement, *χ² (8) = 87.12, p < .0001*, with lower AIC and BIC values as observed in below. In the case of model MB, the comparison between the Full and Level1 models (best and second-best fit, respectively), revealed, as well a better fit, when including the three-way interaction, *χ² (16) = 93.39, p < .0001*. For model MC, the Level1 and Level2 models were identified as the best and second-best fits, respectively. The Level1 model showed an improvement in AIC and BIC compared to the Level2 model, with the likelihood ratio test indicating a significant enhancement in fit, *χ² (8) = 827.31, p < .0001*.

| **Table 4.** Results of the hierarchical comparisons between modelling levels by model. | | | | | | |
| --- | --- | --- | --- | --- | --- | --- |
| **Model** | **Level** | **AIC** | **BIC** | ***χ²*** | **Df.** | **p-value** |
| MA | Full | -23 749 | -23 571 | 87.12 | 8 | < .0001 |
|  | Level1 | -23 678 | -23 559 | 210.39 | 8 | < .0001 |
|  | Level2 | -23 483 | -23 424 | 18.99 | 3 | < .001 |
|  | Null | -23 470 | -23 433 | - | - | - |
| MB | Full | -23 737 | -23 485 | 93.39 | 16 | < .0001 |
|  | Level1 | -23 675 | -23 542 | 211.31 | 8 | < .0001 |
|  | Level2 | -23 480 | -23 406 | 19.84 | 5 | 0.001 |
|  | Null | -23 470 | -23 433 | - | - | - |
| MC | Full | -9 703 | -9 356 | 32.83 | 32 | 0.426 |
|  | Level1 | -9 734 | -9 600 | 827.31 | 8 | < .0001 |
|  | Level2 | -8 923 | -8 843 | 132.80 | 9 | < .0001 |
|  | Null | -8 808 | -8 788 | - | - | - |
| ***Note.*** The comparisons were performed using the likelihood ratio test, AIC and BIC criteria, in a hierarchical fashion. | | | | | | |

# *Contrast analyses models MA and MB*

The results below reported significant three-way interactions in models MA and MB. Table 5 describes congruency contrasts by Bin and target type, while Table 6 presents contrasts between Bins by congruency and target type.

| **Table 5.** Contrasts of congruency (congruent vs. incongruent) by Bin and target type | | | | | | | |
| --- | --- | --- | --- | --- | --- | --- | --- |
| **Model MA** | | | | | | | |
| **Target Type** | **Bin** | **Estimate** | **SE** | **LCL** | **UCL** | **t ratio** | **p value** |
| Social | 1 | -0.014 | 0.007 | -0.03 | 0.00 | -1.87 | 0.063 |
|  | **2** | **-0.025** | **0.007** | **-0.04** | **-0.01** | **-3.28** | **0.001** |
|  | 3 | -0.003 | 0.007 | -0.02 | 0.01 | -0.38 | 0.706 |
|  | 4 | 0.001 | 0.007 | -0.01 | 0.02 | 0.13 | 0.896 |
|  | 5 | 0.010 | 0.007 | -0.01 | 0.02 | 1.27 | 0.205 |
| Non-social | **1** | **0.105** | **0.009** | **0.09** | **0.12** | **11.28** | **< .0001** |
|  | 2 | 0.016 | 0.009 | 0.00 | 0.03 | 1.77 | 0.078 |
|  | 3 | 0.002 | 0.009 | -0.02 | 0.02 | 0.24 | 0.808 |
|  | 4 | -0.002 | 0.009 | -0.02 | 0.02 | -0.17 | 0.864 |
|  | 5 | 0.012 | 0.009 | -0.01 | 0.03 | 1.27 | 0.206 |
| **Model MB** | | | | | | | |
| **Target Type** | **Bin** | **Estimate** | **SE** | **LCL** | **UCL** | **t ratio** | **p value** |
| Face | **1** | **-0.037** | **0.011** | **-0.06** | **-0.01** | **-3.24** | **0.001** |
|  | **2** | **-0.029** | **0.011** | **-0.05** | **-0.01** | **-2.54** | **0.012** |
|  | 3 | 0.000 | 0.011 | -0.02 | 0.02 | 0.00 | 0.998 |
|  | 4 | -0.001 | 0.011 | -0.02 | 0.02 | -0.13 | 0.896 |
|  | 5 | 0.009 | 0.011 | -0.01 | 0.03 | 0.80 | 0.423 |
| Eyes | 1 | 0.004 | 0.010 | -0.02 | 0.02 | 0.41 | 0.679 |
|  | **2** | **-0.021** | **0.010** | **-0.04** | **0.00** | **-2.07** | **0.040** |
|  | 3 | -0.005 | 0.010 | -0.03 | 0.02 | -0.50 | 0.618 |
|  | 4 | 0.003 | 0.010 | -0.02 | 0.02 | 0.30 | 0.766 |
|  | 5 | 0.010 | 0.010 | -0.01 | 0.03 | 0.97 | 0.335 |
| Arrows | **1** | **0.104** | **0.009** | **0.09** | **0.12** | **11.08** | **< .0001** |
|  | 2 | 0.016 | 0.009 | 0.00 | 0.03 | 1.74 | 0.084 |
|  | 3 | 0.002 | 0.009 | -0.02 | 0.02 | 0.24 | 0.813 |
|  | 4 | -0.002 | 0.009 | -0.02 | 0.02 | -0.17 | 0.866 |
|  | 5 | 0.012 | 0.009 | -0.01 | 0.03 | 1.26 | 0.211 |
| ***Note.***  LCL and UCL represent the lower and upper confidence limits, respectively, of the 95% confidence intervals. Significant results are in bold. | | | | | | | |

| **Table 6.** Contrasts between Bins by target type and congruency | | | | | | | |
| --- | --- | --- | --- | --- | --- | --- | --- |
| **Model MA** | | | | | | | |
| **Target Type - congruency** | **Bin**  **Contrast** | **Estimate** | **SE** | **LCL** | **UCL** | **t ratio** | **p value** |
| Social – congruent | **1-2** | **-0.065** | **0.007** | **-0.09** | **-0.04** | **-8.65** | **< .0001** |
|  | **1-3** | **-0.087** | **0.007** | **-0.11** | **-0.07** | **-11.61** | **< .0001** |
|  | **1-4** | **-0.092** | **0.007** | **-0.11** | **-0.07** | **-12.29** | **< .0001** |
|  | **1-5** | **-0.090** | **0.007** | **-0.11** | **-0.07** | **-11.98** | **< .0001** |
|  | **2-3** | **-0.022** | **0.007** | **-0.04** | **0.00** | **-2.96** | **0.028** |
|  | **2-4** | **-0.027** | **0.007** | **-0.05** | **-0.01** | **-3.64** | **0.003** |
|  | **2-5** | **-0.025** | **0.007** | **-0.05** | **0.00** | **-3.32** | **0.009** |
|  | 3-4 | -0.005 | 0.007 | -0.03 | 0.02 | -0.68 | 0.960 |
|  | 3-5 | -0.003 | 0.007 | -0.02 | 0.02 | -0.36 | 0.996 |
|  | 4-5 | 0.002 | 0.007 | -0.02 | 0.02 | 0.32 | 0.998 |
| Social – incongruent | **1-2** | **-0.075** | **0.007** | **-0.10** | **-0.05** | **-10.07** | **< .0001** |
|  | **1-3** | **-0.076** | **0.007** | **-0.10** | **-0.06** | **-10.12** | **< .0001** |
|  | **1-4** | **-0.077** | **0.007** | **-0.10** | **-0.06** | **-10.29** | **< .0001** |
|  | **1-5** | **-0.066** | **0.007** | **-0.09** | **-0.05** | **-8.84** | **< .0001** |
|  | 2-3 | 0.000 | 0.007 | -0.02 | 0.02 | -0.05 | 1.000 |
|  | 2-4 | -0.002 | 0.007 | -0.02 | 0.02 | -0.23 | 0.999 |
|  | 2-5 | 0.009 | 0.007 | -0.01 | 0.03 | 1.23 | 0.733 |
|  | 3-4 | -0.001 | 0.007 | -0.02 | 0.02 | -0.17 | 1.000 |
|  | 3-5 | 0.010 | 0.007 | -0.01 | 0.03 | 1.29 | 0.701 |
|  | 4-5 | 0.011 | 0.007 | -0.01 | 0.03 | 1.46 | 0.591 |
| Non-social - congruent | 1-2 | 0.001 | 0.009 | -0.03 | 0.03 | 0.06 | 1.000 |
|  | 1-3 | 0.002 | 0.009 | -0.02 | 0.03 | 0.20 | 1.000 |
|  | 1-4 | 0.001 | 0.009 | -0.02 | 0.03 | 0.11 | 1.000 |
|  | 1-5 | 0.003 | 0.009 | -0.02 | 0.03 | 0.35 | 0.997 |
|  | 2-3 | 0.001 | 0.009 | -0.02 | 0.03 | 0.14 | 1.000 |
|  | 2-4 | 0.001 | 0.009 | -0.03 | 0.03 | 0.05 | 1.000 |
|  | 2-5 | 0.003 | 0.009 | -0.02 | 0.03 | 0.29 | 0.998 |
|  | 3-4 | -0.001 | 0.009 | -0.03 | 0.02 | -0.09 | 1.000 |
|  | 3-5 | 0.001 | 0.009 | -0.02 | 0.03 | 0.15 | 1.000 |
|  | 4-5 | 0.002 | 0.009 | -0.02 | 0.03 | 0.24 | 0.999 |
| Non-social - incongruent | **1-2** | **-0.088** | **0.009** | **-0.11** | **-0.06** | **-9.45** | **< .0001** |
|  | **1-3** | **-0.101** | **0.009** | **-0.13** | **-0.07** | **-10.84** | **< .0001** |
|  | **1-4** | **-0.105** | **0.009** | **-0.13** | **-0.08** | **-11.34** | **< .0001** |
|  | **1-5** | **-0.090** | **0.009** | **-0.12** | **-0.06** | **-9.66** | **< .0001** |
|  | 2-3 | -0.013 | 0.009 | -0.04 | 0.01 | -1.39 | 0.637 |
|  | 2-4 | -0.018 | 0.009 | -0.04 | 0.01 | -1.89 | 0.327 |
|  | 2-5 | -0.002 | 0.009 | -0.03 | 0.02 | -0.21 | 1.000 |
|  | 3-4 | -0.005 | 0.009 | -0.03 | 0.02 | -0.50 | 0.987 |
|  | 3-5 | 0.011 | 0.009 | -0.01 | 0.04 | 1.18 | 0.764 |
|  | 4-5 | 0.016 | 0.009 | -0.01 | 0.04 | 1.68 | 0.449 |
| **Model MB** | | | | | | | |
| **Target Type - congruency** | **Bin**  **Contrast** | **Estimate** | **SE** | **LCL** | **UCL** | **t ratio** | **p value** |
| Face / congruent | **1-2** | **-0.076** | **0.011** | **-0.11** | **-0.05** | **-6.74** | **< .0001** |
|  | **1-3** | **-0.101** | **0.011** | **-0.13** | **-0.07** | **-8.97** | **< .0001** |
|  | **1-4** | **-0.104** | **0.011** | **-0.14** | **-0.07** | **-9.18** | **< .0001** |
|  | **1-5** | **-0.103** | **0.011** | **-0.13** | **-0.07** | **-9.13** | **< .0001** |
|  | 2-3 | -0.025 | 0.011 | -0.06 | 0.01 | -2.22 | 0.176 |
|  | 2-4 | -0.028 | 0.011 | -0.06 | 0.00 | -2.43 | 0.111 |
|  | 2-5 | -0.027 | 0.011 | -0.06 | 0.00 | -2.39 | 0.124 |
|  | 3-4 | -0.002 | 0.011 | -0.03 | 0.03 | -0.21 | 1.000 |
|  | 3-5 | -0.002 | 0.011 | -0.03 | 0.03 | -0.16 | 1.000 |
|  | 4-5 | 0.001 | 0.011 | -0.03 | 0.03 | 0.05 | 1.000 |
| Face / incongruent | **1-2** | **-0.068** | **0.011** | **-0.10** | **-0.04** | **-6.05** | **< .0001** |
|  | **1-3** | **-0.065** | **0.011** | **-0.10** | **-0.03** | **-5.73** | **< .0001** |
|  | **1-4** | **-0.069** | **0.011** | **-0.10** | **-0.04** | **-6.07** | **< .0001** |
|  | **1-5** | **-0.058** | **0.011** | **-0.09** | **-0.03** | **-5.09** | **< .0001** |
|  | 2-3 | 0.004 | 0.011 | -0.03 | 0.03 | 0.31 | 0.998 |
|  | 2-4 | 0.000 | 0.011 | -0.03 | 0.03 | -0.03 | 1.000 |
|  | 2-5 | 0.011 | 0.011 | -0.02 | 0.04 | 0.96 | 0.874 |
|  | 3-4 | -0.004 | 0.011 | -0.04 | 0.03 | -0.34 | 0.997 |
|  | 3-5 | 0.007 | 0.011 | -0.02 | 0.04 | 0.64 | 0.968 |
|  | 4-5 | 0.011 | 0.011 | -0.02 | 0.04 | 0.98 | 0.862 |
| Eyes / congruent | **1-2** | **-0.056** | **0.010** | **-0.08** | **-0.03** | **-5.43** | **< .0001** |
|  | **1-3** | **-0.075** | **0.010** | **-0.10** | **-0.05** | **-7.35** | **< .0001** |
|  | **1-4** | **-0.083** | **0.010** | **-0.11** | **-0.05** | **-8.07** | **< .0001** |
|  | **1-5** | **-0.079** | **0.010** | **-0.11** | **-0.05** | **-7.69** | **< .0001** |
|  | 2-3 | -0.020 | 0.010 | -0.05 | 0.01 | -1.92 | 0.308 |
|  | 2-4 | -0.027 | 0.010 | -0.06 | 0.00 | -2.64 | 0.066 |
|  | 2-5 | -0.023 | 0.010 | -0.05 | 0.01 | -2.26 | 0.161 |
|  | 3-4 | -0.007 | 0.010 | -0.04 | 0.02 | -0.72 | 0.952 |
|  | 3-5 | -0.003 | 0.010 | -0.03 | 0.02 | -0.34 | 0.997 |
|  | 4-5 | 0.004 | 0.010 | -0.02 | 0.03 | 0.38 | 0.996 |
| Eyes / incongruent | **1-2** | **-0.081** | **0.010** | **-0.11** | **-0.05** | **-7.91** | **< .0001** |
|  | **1-3** | **-0.085** | **0.010** | **-0.11** | **-0.06** | **-8.26** | **< .0001** |
|  | **1-4** | **-0.084** | **0.010** | **-0.11** | **-0.06** | **-8.19** | **< .0001** |
|  | **1-5** | **-0.073** | **0.010** | **-0.10** | **-0.04** | **-7.14** | **< .0001** |
|  | 2-3 | -0.004 | 0.010 | -0.03 | 0.02 | -0.35 | 0.997 |
|  | 2-4 | -0.003 | 0.010 | -0.03 | 0.03 | -0.27 | 0.999 |
|  | 2-5 | 0.008 | 0.010 | -0.02 | 0.04 | 0.77 | 0.938 |
|  | 3-4 | 0.001 | 0.010 | -0.03 | 0.03 | 0.08 | 1.000 |
|  | 3-5 | 0.012 | 0.010 | -0.02 | 0.04 | 1.13 | 0.793 |
|  | 4-5 | 0.011 | 0.010 | -0.02 | 0.04 | 1.05 | 0.832 |
| Arrows – congruent | 1-2 | 0.000 | 0.009 | -0.03 | 0.03 | 0.05 | 1.000 |
|  | 1-3 | 0.002 | 0.009 | -0.02 | 0.03 | 0.19 | 1.000 |
|  | 1-4 | 0.001 | 0.009 | -0.02 | 0.03 | 0.10 | 1.000 |
|  | 1-5 | 0.003 | 0.009 | -0.02 | 0.03 | 0.34 | 0.997 |
|  | 2-3 | 0.001 | 0.009 | -0.02 | 0.03 | 0.14 | 1.000 |
|  | 2-4 | 0.000 | 0.009 | -0.03 | 0.03 | 0.05 | 1.000 |
|  | 2-5 | 0.003 | 0.009 | -0.02 | 0.03 | 0.29 | 0.999 |
|  | 3-4 | -0.001 | 0.009 | -0.03 | 0.03 | -0.09 | 1.000 |
|  | 3-5 | 0.001 | 0.009 | -0.02 | 0.03 | 0.15 | 1.000 |
|  | 4-5 | 0.002 | 0.009 | -0.02 | 0.03 | 0.23 | 0.999 |
| Arrows – incongruent | **1-2** | **-0.087** | **0.009** | **-0.11** | **-0.06** | **-9.29** | **< .0001** |
|  | **1-3** | **-0.100** | **0.009** | **-0.13** | **-0.07** | **-10.65** | **< .0001** |
|  | **1-4** | **-0.105** | **0.009** | **-0.13** | **-0.08** | **-11.15** | **< .0001** |
|  | **1-5** | **-0.089** | **0.009** | **-0.12** | **-0.06** | **-9.49** | **< .0001** |
|  | 2-3 | -0.013 | 0.009 | -0.04 | 0.01 | -1.36 | 0.653 |
|  | 2-4 | -0.017 | 0.009 | -0.04 | 0.01 | -1.85 | 0.346 |
|  | 2-5 | -0.002 | 0.009 | -0.03 | 0.02 | -0.20 | 1.000 |
|  | 3-4 | -0.005 | 0.009 | -0.03 | 0.02 | -0.49 | 0.988 |
|  | 3-5 | 0.011 | 0.009 | -0.01 | 0.04 | 1.17 | 0.771 |
|  | 4-5 | 0.016 | 0.009 | -0.01 | 0.04 | 1.66 | 0.463 |
| ***Note.*** LCL and UCL represent the lower and upper confidence limits, respectively, of the 95% confidence intervals. Significant results are in bold. | | | | | | | |

# *Contrast analyses model MC*

Table 7 presents the results of the congruency contrasts by expression and by bin for model MC. Table 8 reports contrasts between bins by congruency, and Table 9 displays the results of contrasts between expressions by congruency.

| **Table 7.** Contrasts of congruency by expression and by Bins of Model MC. | | | | | | |
| --- | --- | --- | --- | --- | --- | --- |
| **Congruency contrasts by expression** | | | | | | |
| **Expression** | **Estimate** | **SE** | **LCL** | **UCL** | **t ratio** | **p value** |
| anger | **-0.036** | **0.006** | **-0.05** | **-0.02** | **-5.97** | **< .0001** |
| fear | **-0.018** | **0.006** | **-0.03** | **-0.01** | **-2.97** | **0.003** |
| happy | **-0.037** | **0.006** | **-0.05** | **-0.03** | **-6.23** | **< .0001** |
| neutral | **-0.013** | **0.006** | **-0.02** | **0.00** | **-2.19** | **0.029** |
| sad | **-0.028** | **0.006** | **-0.04** | **-0.02** | **-4.67** | **< .0001** |
| **Congruency contrasts by Bin** | | | | | | |
| **Bin** | **Estimate** | **SE** | **LCL** | **UCL** | **t ratio** | **p value** |
| 1 | **-0.091** | **0.006** | **-0.10** | **-0.08** | **-15.36** | **< .0001** |
| 2 | **-0.041** | **0.006** | **-0.05** | **-0.03** | **-6.96** | **< .0001** |
| 3 | **-0.017** | **0.006** | **-0.03** | **-0.01** | **-2.90** | **0.004** |
| 4 | 0.003 | 0.006 | -0.01 | 0.02 | 0.57 | 0.566 |
| 5 | **0.016** | **0.006** | **0.00** | **0.03** | **2.61** | **0.009** |
| ***Note.***  LCL and UCL represent the lower and upper confidence limits, respectively, of the 95% confidence intervals. Significant results are in bold. | | | | | | |

| **Table 8.** Contrasts between Bins by congruency of model MC | | | | | | | | |
| --- | --- | --- | --- | --- | --- | --- | --- | --- |
| **Model MC** | | | | | | | | |
| **Congruency** | **Bin**  **Contrast** | **Estimate** | **SE** | **LCL** | **UCL** | **t ratio** | **p value** | |
| congruent | **1-2** | **-0.099** | **0.006** | **-0.12** | **-0.08** | **-16.67** | **< .0001** | |
|  | **1-3** | **-0.125** | **0.006** | **-0.14** | **-0.11** | **-20.94** | **< .0001** | |
|  | **1-4** | **-0.142** | **0.006** | **-0.16** | **-0.13** | **-23.88** | **< .0001** | |
|  | **1-5** | **-0.139** | **0.006** | **-0.16** | **-0.12** | **-23.38** | **< .0001** | |
|  | **2-3** | **-0.025** | **0.006** | **-0.04** | **-0.01** | **-4.27** | **< .0001** | |
|  | **2-4** | **-0.043** | **0.006** | **-0.06** | **-0.03** | **-7.21** | **< .0001** | |
|  | **2-5** | **-0.040** | **0.006** | **-0.06** | **-0.02** | **-6.71** | **< .0001** | |
|  | **3-4** | **-0.018** | **0.006** | **-0.03** | **0.00** | **-2.94** | **0.027** | |
|  | 3-5 | -0.015 | 0.006 | -0.03 | 0.00 | -2.44 | 0.105 | |
|  | 4-5 | 0.003 | 0.006 | -0.01 | 0.02 | 0.50 | 0.987 | |
| incongruent | **1-2** | **-0.049** | **0.006** | **-0.07** | **-0.03** | **-8.27** | **< .0001** | |
|  | **1-3** | **-0.050** | **0.006** | **-0.07** | **-0.03** | **-8.48** | **< .0001** | |
|  | **1-4** | **-0.047** | **0.006** | **-0.06** | **-0.03** | **-7.94** | **< .0001** | |
|  | **1-5** | **-0.032** | **0.006** | **-0.05** | **-0.02** | **-5.41** | **< .0001** | |
|  | 2-3 | -0.001 | 0.006 | -0.02 | 0.02 | -0.21 | 1.000 | |
|  | 2-4 | 0.002 | 0.006 | -0.01 | 0.02 | 0.32 | 0.998 | |
|  | **2-5** | **0.017** | **0.006** | **0.00** | **0.03** | **2.86** | **0.035** | |
|  | 3-4 | 0.003 | 0.006 | -0.01 | 0.02 | 0.53 | 0.984 | |
|  | **3-5** | **0.018** | **0.006** | **0.00** | **0.03** | **3.07** | **0.018** | |
|  | 4-5 | 0.015 | 0.006 | 0.00 | 0.03 | 2.54 | 0.083 | |
| ***Note.***  LCL and UCL represent the lower and upper confidence limits, respectively, of the 95% confidence intervals. Significant results are in bold. | | | | | | | | |

| **Table 9.** Contrasts between expressions by congruency of model MC | | | | | | | |
| --- | --- | --- | --- | --- | --- | --- | --- |
| **Model MC** | | | | | | | |
| **Congruency** | **contrast** | **Estimate** | **SE** | **LCL** | **UCL** | **t ratio** | **p value** |
| congruent | **anger-fear** | **-0.036** | **0.006** | **-0.05** | **-0.02** | **-5.99** | **< .0001** |
|  | anger-happy | -0.008 | 0.006 | -0.02 | 0.01 | -1.27 | 0.710 |
|  | **anger-neutral** | **-0.028** | **0.006** | **-0.04** | **-0.01** | **-4.73** | **< .0001** |
|  | anger-sad | -0.014 | 0.006 | -0.03 | 0.00 | -2.39 | 0.119 |
|  | **fear-happy** | **0.028** | **0.006** | **0.01** | **0.04** | **4.72** | **< .0001** |
|  | fear-neural | 0.008 | 0.006 | -0.01 | 0.02 | 1.26 | 0.716 |
|  | **fear-sad** | **0.021** | **0.006** | **0.01** | **0.04** | **3.60** | **0.003** |
|  | **happy-neutral** | **-0.021** | **0.006** | **-0.04** | **0.00** | **-3.46** | **0.005** |
|  | happy-sad | -0.007 | 0.006 | -0.02 | 0.01 | -1.12 | 0.799 |
|  | neutral-sad | 0.014 | 0.006 | 0.00 | 0.03 | 2.34 | 0.132 |
| incongruent | **anger-fear** | **-0.018** | **0.006** | **-0.03** | **0.00** | **-2.98** | **0.024** |
|  | anger-happy | -0.009 | 0.006 | -0.03 | 0.01 | -1.53 | 0.544 |
|  | anger-neutral | -0.006 | 0.006 | -0.02 | 0.01 | -0.94 | 0.881 |
|  | anger-sad | -0.006 | 0.006 | -0.02 | 0.01 | -1.08 | 0.817 |
|  | fear-happy | 0.009 | 0.006 | -0.01 | 0.02 | 1.45 | 0.592 |
|  | fear-neural | 0.012 | 0.006 | 0.00 | 0.03 | 2.04 | 0.246 |
|  | fear-sad | 0.011 | 0.006 | 0.00 | 0.03 | 1.90 | 0.316 |
|  | happy-neutral | 0.004 | 0.006 | -0.01 | 0.02 | 0.59 | 0.977 |
|  | happy-sad | 0.003 | 0.006 | -0.01 | 0.02 | 0.45 | 0.992 |
|  | neutral-sad | -0.001 | 0.006 | -0.02 | 0.02 | -0.14 | 1.000 |
| ***Note.***  LCL and UCL represent the lower and upper confidence limits, respectively, of the 95% confidence intervals. Significant results are in bold. | | | | | | | |

# *Four-Bin models: MA and MB*

Four-bin models were computed following the same protocols as those reported in the manuscript with five-bin models. However, we used four 25% bins (e.g., Ülrich et al., 2015, figure 8). The considerations for fixed effects and random parameter structures were the same as for the five-bin models. Similarly, the reported results were obtained using the same R libraries as those used for the five-bin models.

Model MA compared social vs. non-social, whereas model MB compared face vs. eyes. vs. arrows stimuli (Figure 1).

| **A)**  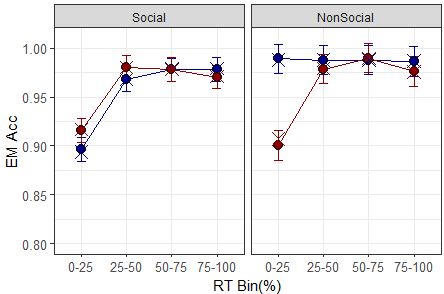 | **B)**  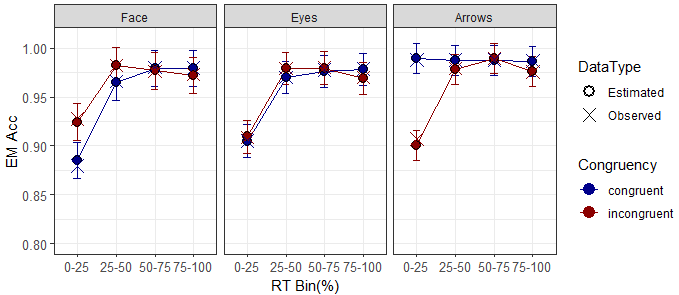 |
| --- | --- |
| **Figure 1.** A) CAF for four bin model MA. B) CAF for four bin model MB. The y-axis represents the estimated marginal means of accuracy rate (EM Acc). The bars represent the 95% confidence intervals of the EM Acc. Observed values are represented by ‘X’ and estimated values by ‘O’. | |

In model MA, the differences between congruent and incongruent trials were noted in Bin 1, with lower accuracy rates for congruent trials in social stimuli, while lower accuracy rates in incongruent trials were noted for non-social stimuli. In model MB, the difference with eyes, although following the same trend as previously observed, was not significant (*p = 0.660*). In contrast, for faces, and arrows, the differences persisted in the same direction as in the model with 5 bins.

| **Table 10.** Contrasts of congruency (congruent vs. incongruent) by Bin and target type of the four bins models | | | | | | | |
| --- | --- | --- | --- | --- | --- | --- | --- |
| **Model MA** | | | | | | | |
| **Target Type** | **Bin** | **Estimate** | **SE** | **LCL** | **UCL** | **t ratio** | **p value** |
| Social | **1** | **-0.020** | **0.007** | **-0.03** | **-0.01** | **-2.84** | **0.005** |
|  | 2 | -0.013 | 0.007 | -0.03 | 0.00 | -1.82 | 0.071 |
|  | 3 | -0.001 | 0.007 | -0.01 | 0.01 | -0.08 | 0.937 |
|  | 4 | 0.008 | 0.007 | -0.01 | 0.02 | 1.11 | 0.267 |
| Non-social | **1** | **0.088** | **0.009** | **0.07** | **0.11** | **10.12** | **< .0001** |
|  | 2 | 0.009 | 0.009 | -0.01 | 0.03 | 1.05 | 0.293 |
|  | 3 | -0.002 | 0.009 | -0.02 | 0.02 | -0.24 | 0.811 |
|  | 4 | 0.010 | 0.009 | -0.01 | 0.03 | 1.17 | 0.242 |
| **Model MB** | | | | | | | |
| **Target Type** | **Bin** | **Estimate** | **SE** | **LCL** | **UCL** | **t ratio** | **p value** |
| Face | **1** | **-0.040** | **0.011** | **-0.06** | **-0.02** | **-3.73** | **< .001** |
|  | 2 | -0.017 | 0.011 | -0.04 | 0.00 | -1.60 | 0.113 |
|  | 3 | 0.003 | 0.011 | -0.02 | 0.02 | 0.26 | 0.793 |
|  | 4 | 0.007 | 0.011 | -0.01 | 0.03 | 0.62 | 0.536 |
| Eyes | 1 | -0.004 | 0.010 | -0.02 | 0.01 | -0.44 | 0.660 |
|  | 2 | -0.009 | 0.010 | -0.03 | 0.01 | -0.98 | 0.330 |
|  | 3 | -0.003 | 0.010 | -0.02 | 0.02 | -0.34 | 0.734 |
|  | 4 | 0.009 | 0.010 | -0.01 | 0.03 | 0.93 | 0.356 |
| Arrows | **1** | **0.088** | **0.009** | **0.07** | **0.11** | **9.97** | **< .0001** |
|  | 2 | 0.009 | 0.009 | -0.01 | 0.03 | 1.04 | 0.301 |
|  | 3 | -0.002 | 0.009 | -0.02 | 0.02 | -0.24 | 0.814 |
|  | 4 | 0.010 | 0.009 | -0.01 | 0.03 | 1.16 | 0.247 |
| ***Note.***  LCL and UCL represent the lower and upper confidence limits, respectively, of the 95% confidence intervals. Significant results are in bold. | | | | | | | |

The contrasts between Bins across congruency levels showed similar patterns to those observed with five-bin models. In model MA, social targets exhibited a significant reduction in accuracy in bin 1 compared to the other bins in both congruent and incongruent trials, whereas non-social targets showed a reduction in accuracy only in incongruent trials. Similarly, in model MB, both congruency levels showed reduced accuracy in the first bin compared to the rest with face and eye stimuli, whereas arrows showed a reduction only in incongruent trials.

| **Table 11.** Contrasts of Bin by target type and congruency of the four bins models | | | | | | | |
| --- | --- | --- | --- | --- | --- | --- | --- |
| **Model MA** | | | | | | | |
| **Target Type - congruency** | **Bin**  **Contrast** | **Estimate** | **SE** | **LCL** | **UCL** | **t ratio** | **p value** |
| Social – congruent | **1-2** | **-0.071** | **0.007** | **-0.09** | **-0.05** | **-10.16** | **< .0001** |
|  | **1-3** | **-0.082** | **0.007** | **-0.10** | **-0.06** | **-11.60** | **< .0001** |
|  | **1-4** | **-0.082** | **0.007** | **-0.10** | **-0.06** | **-11.69** | **< .0001** |
|  | 2-3 | -0.010 | 0.007 | -0.03 | 0.01 | -1.44 | 0.475 |
|  | 2-4 | -0.011 | 0.007 | -0.03 | 0.01 | -1.52 | 0.425 |
|  | 3-4 | -0.001 | 0.007 | -0.02 | 0.02 | -0.08 | 1.000 |
| Social – incongruent | **1-2** | **-0.064** | **0.007** | **-0.08** | **-0.05** | **-9.14** | **< .0001** |
|  | **1-3** | **-0.062** | **0.007** | **-0.08** | **-0.04** | **-8.84** | **< .0001** |
|  | **1-4** | **-0.054** | **0.007** | **-0.07** | **-0.04** | **-7.73** | **< .0001** |
|  | 2-3 | 0.002 | 0.007 | -0.02 | 0.02 | 0.29 | 0.991 |
|  | 2-4 | 0.010 | 0.007 | -0.01 | 0.03 | 1.41 | 0.497 |
|  | 3-4 | 0.008 | 0.007 | -0.01 | 0.03 | 1.11 | 0.682 |
| Non-social - congruent | 1-2 | 0.001 | 0.009 | -0.02 | 0.02 | 0.15 | 0.999 |
|  | 1-3 | 0.001 | 0.009 | -0.02 | 0.02 | 0.14 | 0.999 |
|  | 1-4 | 0.003 | 0.009 | -0.02 | 0.03 | 0.30 | 0.990 |
|  | 2-3 | 0.000 | 0.009 | -0.02 | 0.02 | -0.01 | 1.000 |
|  | 2-4 | 0.001 | 0.009 | -0.02 | 0.02 | 0.15 | 0.999 |
|  | 3-4 | 0.001 | 0.009 | -0.02 | 0.02 | 0.16 | 0.999 |
| Non-social - incongruent | **1-2** | **-0.078** | **0.009** | **-0.10** | **-0.06** | **-8.92** | **< .0001** |
|  | **1-3** | **-0.089** | **0.009** | **-0.11** | **-0.07** | **-10.22** | **< .0001** |
|  | **1-4** | **-0.075** | **0.009** | **-0.10** | **-0.05** | **-8.65** | **< .0001** |
|  | 2-3 | -0.011 | 0.009 | -0.03 | 0.01 | -1.30 | 0.562 |
|  | 2-4 | 0.002 | 0.009 | -0.02 | 0.03 | 0.27 | 0.993 |
|  | 3-4 | 0.014 | 0.009 | -0.01 | 0.04 | 1.57 | 0.398 |
| **Model MB** | | | | | | | |
| **Target Type - congruency** | **Bin**  **Contrast** | **Estimate** | **SE** | **LCL** | **UCL** | **t ratio** | **p value** |
| Face / congruent | **1-2** | **-0.080** | **0.011** | **-0.11** | **-0.05** | **-7.54** | **< .0001** |
|  | **1-3** | **-0.095** | **0.011** | **-0.12** | **-0.07** | **-8.90** | **< .0001** |
|  | **1-4** | **-0.094** | **0.011** | **-0.12** | **-0.07** | **-8.84** | **< .0001** |
|  | 2-3 | -0.014 | 0.011 | -0.04 | 0.01 | -1.36 | 0.527 |
|  | 2-4 | -0.014 | 0.011 | -0.04 | 0.01 | -1.30 | 0.563 |
|  | 3-4 | 0.001 | 0.011 | -0.03 | 0.03 | 0.06 | 1.000 |
| Face / incongruent | **1-2** | **-0.058** | **0.011** | **-0.09** | **-0.03** | **-5.41** | **< .0001** |
|  | **1-3** | **-0.052** | **0.011** | **-0.08** | **-0.02** | **-4.91** | **< .0001** |
|  | **1-4** | **-0.048** | **0.011** | **-0.08** | **-0.02** | **-4.50** | **< .0001** |
|  | 2-3 | 0.005 | 0.011 | -0.02 | 0.03 | 0.50 | 0.959 |
|  | 2-4 | 0.010 | 0.011 | -0.02 | 0.04 | 0.91 | 0.797 |
|  | 3-4 | 0.004 | 0.011 | -0.02 | 0.03 | 0.41 | 0.976 |
| Eyes / congruent | **1-2** | **-0.065** | **0.010** | **-0.09** | **-0.04** | **-6.73** | **< .0001** |
|  | **1-3** | **-0.071** | **0.010** | **-0.10** | **-0.05** | **-7.43** | **< .0001** |
|  | **1-4** | **-0.073** | **0.010** | **-0.10** | **-0.05** | **-7.59** | **< .0001** |
|  | 2-3 | -0.007 | 0.010 | -0.03 | 0.02 | -0.70 | 0.898 |
|  | 2-4 | -0.008 | 0.010 | -0.03 | 0.02 | -0.86 | 0.828 |
|  | 3-4 | -0.002 | 0.010 | -0.03 | 0.02 | -0.16 | 0.999 |
| Eyes / incongruent | **1-2** | **-0.070** | **0.010** | **-0.09** | **-0.04** | **-7.27** | **< .0001** |
|  | **1-3** | **-0.070** | **0.010** | **-0.10** | **-0.05** | **-7.33** | **< .0001** |
|  | **1-4** | **-0.060** | **0.010** | **-0.08** | **-0.03** | **-6.22** | **< .0001** |
|  | 2-3 | -0.001 | 0.010 | -0.03 | 0.02 | -0.06 | 1.000 |
|  | 2-4 | 0.010 | 0.010 | -0.01 | 0.03 | 1.05 | 0.722 |
|  | 3-4 | 0.011 | 0.010 | -0.01 | 0.04 | 1.11 | 0.686 |
| Arrows – congruent | 1-2 | 0.001 | 0.009 | -0.02 | 0.02 | 0.15 | 0.999 |
|  | 1-3 | 0.001 | 0.009 | -0.02 | 0.02 | 0.14 | 0.999 |
|  | 1-4 | 0.003 | 0.009 | -0.02 | 0.03 | 0.29 | 0.991 |
|  | 2-3 | 0.000 | 0.009 | -0.02 | 0.02 | -0.01 | 1.000 |
|  | 2-4 | 0.001 | 0.009 | -0.02 | 0.02 | 0.15 | 0.999 |
|  | 3-4 | 0.001 | 0.009 | -0.02 | 0.02 | 0.16 | 0.999 |
| Arrows – incongruent | **1-2** | **-0.078** | **0.009** | **-0.10** | **-0.05** | **-8.79** | **< .0001** |
|  | **1-3** | **-0.089** | **0.009** | **-0.11** | **-0.07** | **-10.07** | **< .0001** |
|  | **1-4** | **-0.075** | **0.009** | **-0.10** | **-0.05** | **-8.51** | **< .0001** |
|  | 2-3 | -0.011 | 0.009 | -0.03 | 0.01 | -1.28 | 0.576 |
|  | 2-4 | 0.002 | 0.009 | -0.02 | 0.03 | 0.27 | 0.993 |
|  | 3-4 | 0.014 | 0.009 | -0.01 | 0.04 | 1.55 | 0.409 |
| ***Note.*** LCL and UCL represent the lower and upper confidence limits, respectively, of the 95% confidence intervals. Significant results are in bold. Significant results are in bold. | | | | | | | |

Overall, the interaction plot (Figure 2) revealed that the patterns align with reported trends across five bins, especially concerning the differences between stimulus types. Even though the dissociation with eye stimuli did not reach significance, there is a clear distinction when compared to arrows in the first bin. This change could be explained by the fact that with five bins, the distribution is divided in a more nuanced manner, making the range for faster responses more specific. In contrast, a four-bin CAF might mix information between faster and middle response latencies.

| **A)** | |
| --- | --- |
| 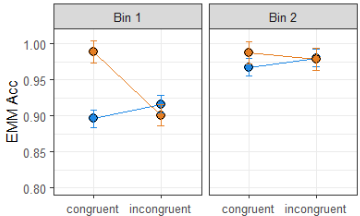 | 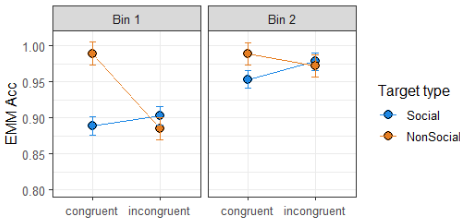 |
| **B)** | |
| 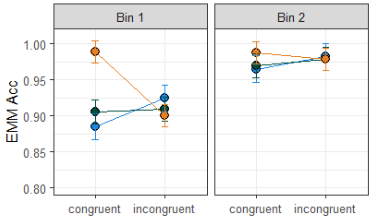 | 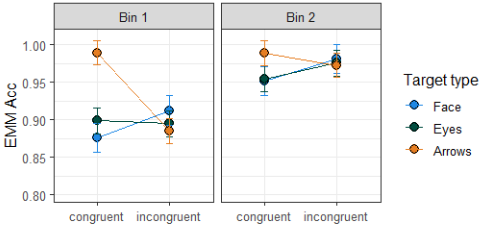 |
| **Figure 2.** Interaction plot for the first two bins of congruency levels by target type. The left figures display the 4-bin models, while the right figures are the 5-bin models. A) Model MA compares social versus non-social stimuli. B) Model MB compares face, eyes, and arrows stimuli. | |

**REFERENCES**

Ambrosi, S., Servant, M., Blaye, A., & Burle, B. (2019). Conflict processing in kindergarten children: New evidence from distribution analyses reveals the dynamics of incorrect response activation and suppression. Journal of experimental child psychology, 177, 36–52. https://doi.org/10.1016/j.jecp.2018.06.006

Barr, D. J. (2013). Random effects structure for testing interactions in linear mixed-effects models. Frontiers in Psychology, 4, Article 328. https://doi.org/10.3389/fpsyg.2013.00328

Barr D. J., Levy R., Scheepers C., Tily H. J. (2013). Random effects structure for confirmatory hypothesis testing: Keep it maximal. J. Mem. Lang. 68, 255–278 10.1016/j.jml.2012.11.001

Bates, D., Mächler, M., Bolker, B., & Walker, S. (2015). Fitting Linear Mixed-Effects Models Using lme4. Journal of Statistical Software, 67(1), 1–48. https://doi.org/10.18637/jss.v067.i01

Bonventre, S. & Marotta, A. (2023). Is the reversed congruency effect unique to the eye-gaze? Investigating the effects of finger pointing, eye-gaze and arrows stimuli on spatial interference. Front. Cognit. 2: 1135435.doi:10.3389/fcogn.2023.1135435

Brooks, M.E., K. Kristensen, K.J. van Benthem, A. Magnusson, C.W. Berg, A. Nielson, H.J. Skaug, M. Maechler, and B.M. Bolker. 2017. “glmmTMB Balances Speed and Flexibility among Packages for Zero-Inflated Generalized Linear Mixed Modeling.” The R Journal 9: 378–400.

Dalmaso, M., Galfano, G., & Castelli, L. (2023). Are eyes special? Gaze, but not pointing gestures, elicits a reversed congruency effect in a spatial Stroop task. Atten Percept Psychophys. https://doi.org/10.3758/s13414-023-02774-6

Ferrari SLP, Cribari-Neto F (2004) Beta regression for modelling rates and proportions. Journal of Applied Statistics 31: 799–815.

Geisser, S. (1975). The predictive sample reuse method with applications. Journal of the American Statistical Association, 70(350): 320–328.

Hemmerich, K., Narganes-Pineda, C., Marotta, A., Martín-Arévalo, E., Jiménez, L., & Lupiáñez, J. (2022). Gaze elicits social and nonsocial attentional orienting: An interplay of shared and unique conflict processing mechanisms. Journal of Experimental Psychology: Human Perception and Performance, 48(8), 824–841. <https://doi.org/10.1037/xhp0001015>

Marotta, A., Lupiáñez, J., Román-Caballero, R., Narganes-Pineda, C., & Martín-Arévalo, E. (2019). Are eyes special? Electrophysiological and behavioural evidence for a dissociation between eye-gaze and arrows attentional mechanisms. Neuropsychologia, 129, 146–152. https://doi.org/10.1016/j.neuropsychologia.2019.03.017

Marotta, A., Román-Caballero, R., & Lupiáñez, J. (2018). Arrows don’t look at you: Qualitatively different attentional mechanisms triggered by gaze and arrows. Psychonomic Bulletin & Review, 25(6), 2254–2259. <https://doi.org/10.3758/s13423-018-1457-2>

Meteyard, L., & Davies, R. A. I. (2020). Best practice guidance for linear mixed-effects models in psychological science. Journal of Memory and Language, 112, Article 104092. <https://doi.org/10.1016/j.jml.2020.104092>

Ratcliff R. (1993). Methods for dealing with reaction time outliers. Psychological bulletin, 114(3), 510–532. https://doi.org/10.1037/0033-2909.114.3.510

Schielzeth, H. and Nakagawa, S. (2013) Nested by Design: Model Fitting and Interpretation in a Mixed Model Era. Methods in Ecology and Evolution, 4, 14-24. https://doi.org/10.1111/j.2041-210x.2012.00251.x

Ulrich, R., & Miller, J. (1994). Effects of truncation on reaction time analysis. Journal of experimental psychology. General, 123(1), 34–80. <https://doi.org/10.1037//0096-3445.123.1.34>

Ulrich, R., Schröter, H., Leuthold, H., & Birngruber, T. (2015). Automatic and controlled stimulus processing in conflict tasks: Superimposed diffusion processes and delta functions. Cognitive psychology, 78, 148–174. https://doi.org/10.1016/j.cogpsych.2015.02.005
